# Empirical Validation of SNR-Dependent Deep Learning Architectures for Label-Free Super-Resolution in Optical Microscopy

**DOI:** 10.3390/nano16201334

**Published:** 2026-10-21

**Authors:** Shiraz S. Kaderuppan, Jonathan D. Mar, Andrew C. Irvine, Anurag Sharma, Muhammad Ramadan Saifuddin, Wai Leong Eugene Wong, Wai Lok Woo

**Affiliations:** 1 Newcastle University in Singapore, 1 Punggol Coast Road, Block E1, Level 2, Singapore 828608, Singapore; s.s.o.kaderuppan@newcastle.ac.uk (S.S.K.); ramadan.saifuddin@newcastle.ac.uk (M.R.S.); 2 Faculty of Science, Agriculture and Engineering (SAgE), Newcastle University, Newcastle upon Tyne NE1 7RU, UK; 3 Department of Electrical, Computer, and Biomedical Engineering, Toronto Metropolitan University, Toronto, ON M5B 2K3, Canada; jdmar@torontomu.ca; 4 Cavendish Laboratory, University of Cambridge, JJ Thomson Avenue, Cambridge CB3 0US, UK; aci20@cam.ac.uk; 5 Engineering Cluster, Singapore Institute of Technology, 10 Dover Drive, Singapore 138683, Singapore; eugene.wong@singaporetech.edu.sg; 6 School of Computer Science, Northumbria University, Newcastle upon Tyne NE1 8ST, UK; wailok.woo@northumbria.ac.uk

**Keywords:** deep neural networks in microscopy, *in silico*/computational super-resolution microscopy, non-fluorescent nanoscopy, phase-dependent optical nanoscopy

## Abstract

Optical microscopy is widely used across scientific, industrial, and clinical settings, yet its lateral resolution is fundamentally limited by diffraction (down to ~200 nm in 400 nm visible light). While super-resolution techniques can overcome this barrier, most existing approaches rely on specialised hardware or fluorescent labelling, restricting their accessibility in routine microscopy workflows. As such, past research studies (including our own) have focused on developing deep neural network (DNN) architectures capable of super-resolving optical micrographs. In the present context, two of our previously developed DNN architectures, termed O-Net and Θ-Net, are systematically evaluated using a novel custom-fabricated optical test target containing nanoscale features independently calibrated by atomic force microscopy (AFM). The results indicate that both architectures surpass the diffraction-limited resolution of the optical system employing halogen illumination (peak λ_em_ = 619.4 nm; $\bar{\lambda_{\mathrm{em}}}$ = 612.2 nm), resolving features down to 170 nm in PCM and 200 nm in DIC (at least). Additionally, the models exemplify a *complementary* SNR-dependent behaviour—O-Net achieves superior performance under high-SNR conditions, while Θ-Net demonstrates enhanced robustness when SNR is reduced. These findings have important implications in computational super-resolution microscopy, highlighting the potential applicability of the assayed model architectures under differing image acquisition protocols.

## 1. Introduction

Super-resolution optical microscopy (SROM) encompasses approaches developed to overcome the spatial-resolution limitations imposed by optical diffraction. The classical Rayleigh criterion (which describes the theoretical limit of resolution for an optical microscope) has constrained optical microscopy for over a century [1], and may be expressed by Equation (1) as follows [2]:

Rayleigh Criterion

(1) $$d_{x,\;y}=\frac{0.61\lambda }{{\mathrm{NA}}_{\mathrm{obj}}}\;(\mathrm{for}\;\mathrm{fluorescence})\;\mathrm{or}\;\frac{1.22\lambda }{{\mathrm{NA}}_{\mathrm{cond}}+{\mathrm{NA}}_{\mathrm{obj}}}(\mathrm{for}\;\mathrm{brightfield}),$$

where λ, NA_obj_ and NA_cond_ refer to the wavelength of the light source, and the numerical apertures of the objective and condenser lenses used respectively.

A range of optical strategies have subsequently been developed, aimed at extending the attainable resolution, which include confocal laser scanning microscopy (CLSM) [3], 4Pi microscopy [4], and near-field scanning optical microscopy (NSOM) [5]. The emergence of *fluorescence nanoscopy*, particularly stimulated emission depletion (STED) [6] and single-molecule localisation approaches such as PALM [7] and STORM [8], established the feasibility of routinely accessing sub-100 nm length scales. More recently, MINSTED has demonstrated localisation precision down to σ = 4.7 Å [9], exemplifying the resolution enhancement realised through optical fluorescence nanoscopy in surpassing the Rayleigh diffraction limit by up to three orders of magnitude and transcending SEM approaches [10]. However, these techniques generally depend on fluorescent labels and therefore remain subject to limitations associated with photobleaching, phototoxicity and perturbation, fluorescence cross-talk, and non-specific or incomplete labelling [11]. Even fluorescence approaches (such as *multiphoton microscopy* [12]) generally utilise a comparatively restricted set of endogenous fluorophores, such as NADH [13].

The aforementioned limitations have fuelled increasing interest in **label-free non-fluorescence optical imaging**, particularly *quantitative phase imaging* (QPI), in which intrinsic optical phase or refractive-index variations provide contrast without exogenous labels. QPI has subsequently developed into a broad family of techniques capable of providing quantitative information on cellular morphology, biomass distribution, and dynamics while avoiding several limitations associated with fluorescence imaging ([14,15,16]). The integration of artificial intelligence (AI) with QPI has further expanded this capability to include image enhancement, reconstruction, segmentation, and image translation [14]. Concurrently, computational approaches have also demonstrated that deep neural networks (DNNs) are capable of inferring higher-resolution information from conventional fluorescence optical micrographs, leading to the development of DNNs such as ANNA-PALM [17], PCSR [18], Deep-STORM [19] and FID-STORM [20] for computational fluorescence super-resolution (SR), while O-Net [21], Θ-Net [22] and RFGANM [23] have been developed for label-free computational SR utilising *non-fluorescent* microscopy modalities, such as brightfield (BF), differential interference contrast (DIC), and phase-contrast microscopy (PCM). Optical strategies adopting solid immersion lenses [24] and microspheres [25] have also been investigated for *non-fluorescent optical nanoscopy*, with a broader discussion provided in Ref. [26] for the interested reader.

Despite the aforementioned advances in DNN-mediated SR microscopy, a critical distinction must be made between **generating visually enhanced images and empirically demonstrating that previously unresolved spatial information has actually been recovered**. This distinction becomes particularly important for learning-based image restoration, since high-frequency structures that are weakly represented or absent in the measured image can be confounded with noise or inferred from statistical priors. Consequently, DNN-based restoration can introduce, suppress, or alter structural features, resulting in network hallucination artifacts [21]. Physics-informed networks (PINs) may provide an important complementary direction for feature reconstruction, particularly for problems in which the underlying physical constraints can be incorporated to improve the fidelity of image processing tasks (such as denoising, phase retrieval, deconvolution, or aberration correction) [27,28]. However, physical consistency alone does not establish that a super-resolved image contains experimentally recoverable information beyond the spatial-frequency support of the imaging system. Indeed, for a conventional diffraction-limited optical system, spatial frequencies that are not transmitted by the optical transfer function are not directly encoded in the measurement; hence, imposing the forward physics of the same imaging system cannot (by itself) demonstrate recovery of genuinely absent spatial information. Spatial frequencies that are masked by inherent noise in the imaging system may manifest similar constraints when a PIN is employed. A DNN/GAN, however, may generate plausible higher-frequency structures based on mapping of learnt features/priors and/or mathematical relationships between the low-resolution and high-resolution images in the training image dataset, making it essential to distinguish between feature mapping and true resolution enhancement. In this respect, uncertainty-aware approaches may provide a further means of identifying regions or structures for which the model has limited confidence [29], but ultimately the claim that previously unresolved structures have been recovered requires independent experimental validation against a spatially characterised and empirically calibrated reference standard.

This issue becomes particularly pertinent in phase-dependent microscopy modalities (e.g., PCM and DIC), where the absence of an empirically calibrated nanoscale reference standard (with features < 200 nm) commercially has made it generally difficult to distinguish the genuine recovery of unresolved structures from model-dependent enhancement. Accordingly, a specially fabricated and calibrated test target spanning a broad range of feature dimensions provides a more direct means of determining the smallest structures that can be reliably recovered and of quantifying the effective resolution enhancement of a computational model. Such a target sample would thus also address a related secondary issue—the empirical determination of the *actual* resolution enhancement factor attained through the employment of a DNN model. Claims of resolution enhancements beyond the Rayleigh limit would require validation against an independently characterised and calibrated spatial standard, rather than visual inspection alone or comparison with network-generated SR reference images. This would also concur with past studies promulgating rigorous benchmarking of microscopy restoration methods, through an increased emphasis on controlled imaging conditions, quantitative performance assessment, and experimentally meaningful reference data [30].

A second unresolved concern relates to the potential role of other image acquisition confounders (such as noise) on the SR output of a DNN model, particularly for PCM and DIC microscopy, where the input image is itself governed by the optical transfer characteristics and phase-dependent contrast mechanisms of the microscope. Although previous computational studies have demonstrated super-resolution from such modalities [21,22,23], systematic assessment of how image quality factors [particularly the input (**Source**) image *signal-to-noise ratio* (SNR)] affect the suitability of different network architectures has remained comparatively limited. Prior published work [31,32] has investigated the influence of SNR on DNN-mediated SR fluorescence microscopy, although this aspect has not been comparably established for computational SR *phase-dependent* microscopy approaches. This represents an important consideration as sub-micron structures occupy higher spatial frequencies and may consequently be interpreted by a trained network as *pseudo-noise* [21]. Depending on the DNN architecture and the model training conditions, such behaviour can result in either the suppression of genuine structures or the generation of non-existent features, resulting in network hallucination artifacts. Establishing the SNR conditions under which a particular DNN model architecture can reliably recover spatial information is therefore essential for determining its practical operating range.

In light of these considerations, the present work makes two principal contributions as follows:

Firstly, we determine the optimal network architecture, among our two previously developed and published architectures (O-Net [21] and Θ-Net [22]), as well as U-Net [33], for super-resolution of label-free phase-dependent optical micrographs as a function of the SNR of the input (**Source**) image. To our knowledge, the dependence of DNN architecture selection on the input image SNR has not been previously investigated and/or established for these phase-dependent microscopy SR DNN architectures.

Secondly, we empirically validate the super-resolution capability of our trained O-Net and Θ-Net models using a novel custom-fabricated, AFM-calibrated test target containing features spanning from 20 nm to 6 μm. This enables the resolution limit and corresponding resolution-enhancement factor of the models adopting each network architecture to be quantified experimentally, rather than inferred solely from visual appearance or network-generated reference images. 

The methodology presented here, together with the corresponding quantitative assessment of resolution and SNR dependence, therefore extends our previous work [21,22] and constitutes an extrapolation of the methodology introduced in our pre-print article [34]. The subsequent sections of this manuscript describe the experimental test target, image acquisition and processing procedures, network architectures, and quantitative frameworks used to establish the effective SR performance of O-Net and Θ-Net in phase-dependent optical microscopy.

## 2. Methodology

### 2.1. Image Acquisition for Model Training and Validation Datasets

As was described in our previously published studies [21,22,34], identical model training image datasets were used in the present context, comprising PCM (Zernike) and DIC micrographs acquired from 50 different samples (9 plant sections, 30 animal tissues and 11 protists). The hardware setup consisted of a Leica N PLAN L 20×/0.4 Corr Ph1 objective (Leica P/N: 506058) and a Leica HCX PL Fluotar L 40×/0.60 Corr Ph2 objective (Leica P/N: 506203) mounted on a Leica DM4000M microscope and with a CMOS camera (RisingCam® E3ISPM12000KPA, Chengdu Ruishen Technology Co., LTD, Chengdu, Sichuan, China) with a pixel size of 1.85 μm × 1.85 μm and an EK 14 Mot motorised stage (Märzhäuser Wetzlar GmbH & Co. KG, Wetzlar, Germany). A 0.7× C-mount was coupled with an empirically validated tube magnification factor of 1.12× and was present in the optical train of the microscope prior to image acquisition by the camera sensor (for all of the objectives utilised in the present study). In addition to this, a Leica S1 (0.90 NA) condenser top (Leica P/N: 11505150) was used for the acquisition of all raw PCM and DIC images in the present study. The 20×/0.40 objective was used for acquiring low-resolution (LR) images, while the 40×/0.60 objective was used to obtain high-resolution (HR) images. Due to the differences in the field of view of these two objectives, an intermediate magnification changer (with 2.0× magnification) was used together with the 20×/0.40 objective. Similar regions of interest (RoIs) were imaged of the samples under both PCM and DIC microscopy via a self-developed desktop app coded in C# .NET 4.7, with 3D Z-stacks acquired of thicker specimens necessitating multiple focal-plane capture across the entire RoI for the computational reconstruction of a 2D *extended depth-of-field* (EDF) image within Helicon Focus 8.1.0 Pro (© Helicon Soft Ltd., 2000, Kharkiv, Ukraine). The acquired/EDF images were then registered in MATLAB R2020a (© 1984–2020, The MathWorks, Inc., Natick, MA, USA), layered and cropped within Corel® PHOTO-PAINT™ X7 (© 2015 Corel Corporation, Ottawa, ON, Canada), and subsequently split into 600 px × 600 px image tiles for concatenation into LR (**Source**)-HR (**Expected**/target) image pairs, prior to image downscaling (to a 256 px × 512 px format) in MATLAB R2020a (© 1984–2020, The MathWorks, Inc., Natick, MA, USA) [22]. These image tile pairs (a total of 3944 image pairs per imaging modality employed, comprising 2564 animal, 792 plant and 588 protists) were then converted into NumPy arrays for training the individual nodes of the proposed Θ-Net network in Python 3.9 [22]. The distribution of chosen sample types (across plant and animal tissues, as well as protists) ensures that the trained models were not overfitted to a particular sample type (e.g., animal tissue) and generalised across a wide range of different features. In addition, the trained models were validated on image datasets to which they were not exposed previously, alleviating potential concerns arising from network overfitting. These validation datasets were acquired from an additional 6 slides (2 each of animal tissues, plant sections and protist samples) under the same imaging conditions as the training dataset, and subsequently used to generate 610 image tile pairs (256 px × 512 px) comprising 184 animal, 184 plant and 242 protist samples, thereby constituting an independent image dataset for validation purposes.

### 2.2. DNN Architectures and Model Training

**Figure 1:**
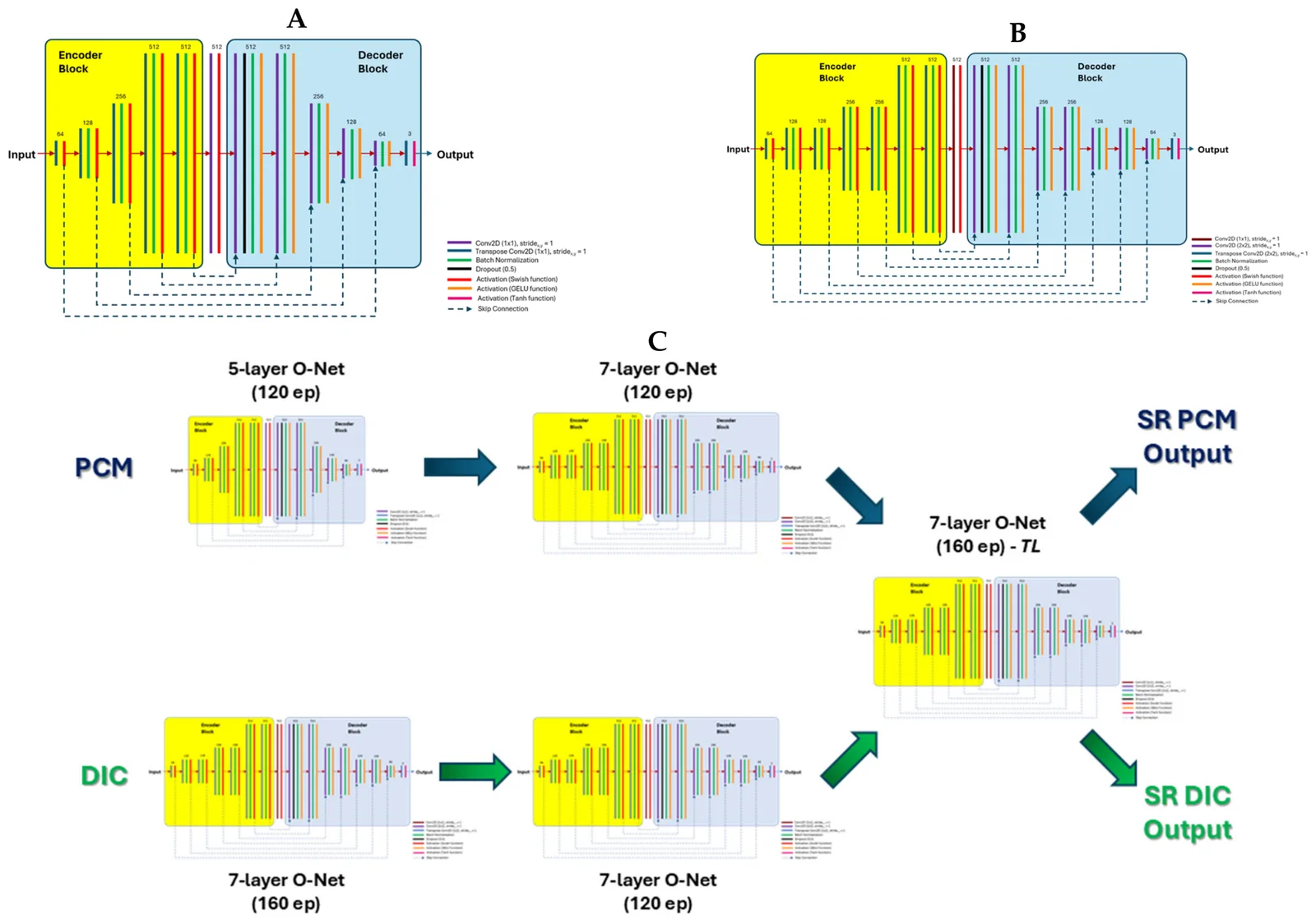
O-Net DNN architectures, employing either a 5-layer (**A**) or 7-layer (**B**) network. The 5-layer O-Net architecture employs same-size image restoration, while the 7-layer O-Net employs spatial upsampling followed by downsampling [both network architectures sharing a similar input/output tensor size of 256 × 256 × 3 (scale factor = 1)]. As highlighted in Ref. [21], each O-Net architecture contains skip-connections (*concatenations*) linking transposed convolution layers (in the encoder block) to convolution layers (in the decoder block). Concatenation of multiple O-Net frameworks (*nodes*) results in the formation of a Θ-Net model framework (an example of which is depicted in (**C**), as described in Ref. [22] and in the current context. In the present study, both the DIC and PCM Θ-Net model training pipelines utilised differing O-Net models, with the exception of the 3rd (and final) node—a common O-Net model trained on *both* the DIC and PCM image datasets (i.e., *transfer learning*). Figures are adapted from Refs. [22,34].

For the purposes of this study, the DNN models adopting the O-Net and Θ-Net architectures were trained on a workstation equipped with an Intel^®^ Core™ i9-10920X CPU, 128GB RAM and an NVIDIA RTX™ 3090 GPU (© 2022, NVIDIA Corporation, Santa Clara, CA, USA). The model architectures used were similar to those described in [22], i.e., a single-node 5-layer/7-layer O-Net architecture (for the O-Net models) or a triple-node O-Net architecture (for the Θ-Net models) as the *generator* network in a generative adversarial network (GAN), with Figure 1 depicting the details of each of these DNN architectures.

For the U-Net models, the model architecture as described in [21] (a 5-layer U-Net architecture) was followed. Additionally, all the models adopting the various DNN architectures (O-Net, Θ-Net and U-Net) were trained on the same image dataset, and subsequently validated using identical photomicrographs acquired of the same AFM-calibrated test target sample (prepared as described in the following section). For auditing purposes, the hyperparameters (as described in Table 1) were utilised for both the O-Net and Θ-Net model training runs (the detailed architectures for each of these networks being described in Supplementary Figures S3–S5):

### 2.3. Evaluation/Testing of Trained Models

**Figure 2:**
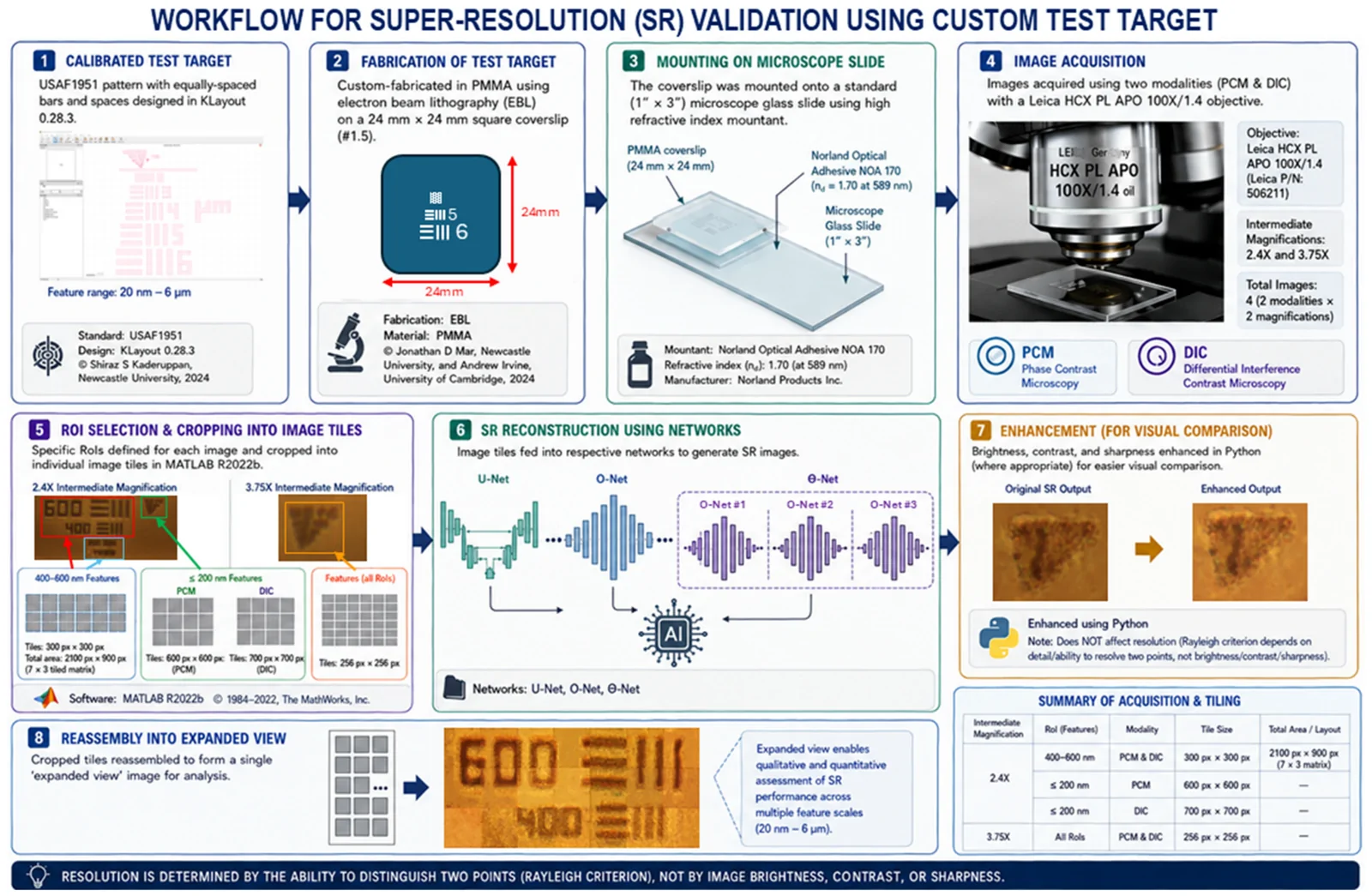
The experimental evaluation pipeline as utilised in the present study employing identical input images for the U-Net, O-Net and Θ-Net models. The outputs derived from each model (being different) may then be compared against one another, providing a means for verifying model performance. Figure generated using ChatGPT (GPT-6) from the previously described procedure, and subsequently modified.

**Figure 4:**
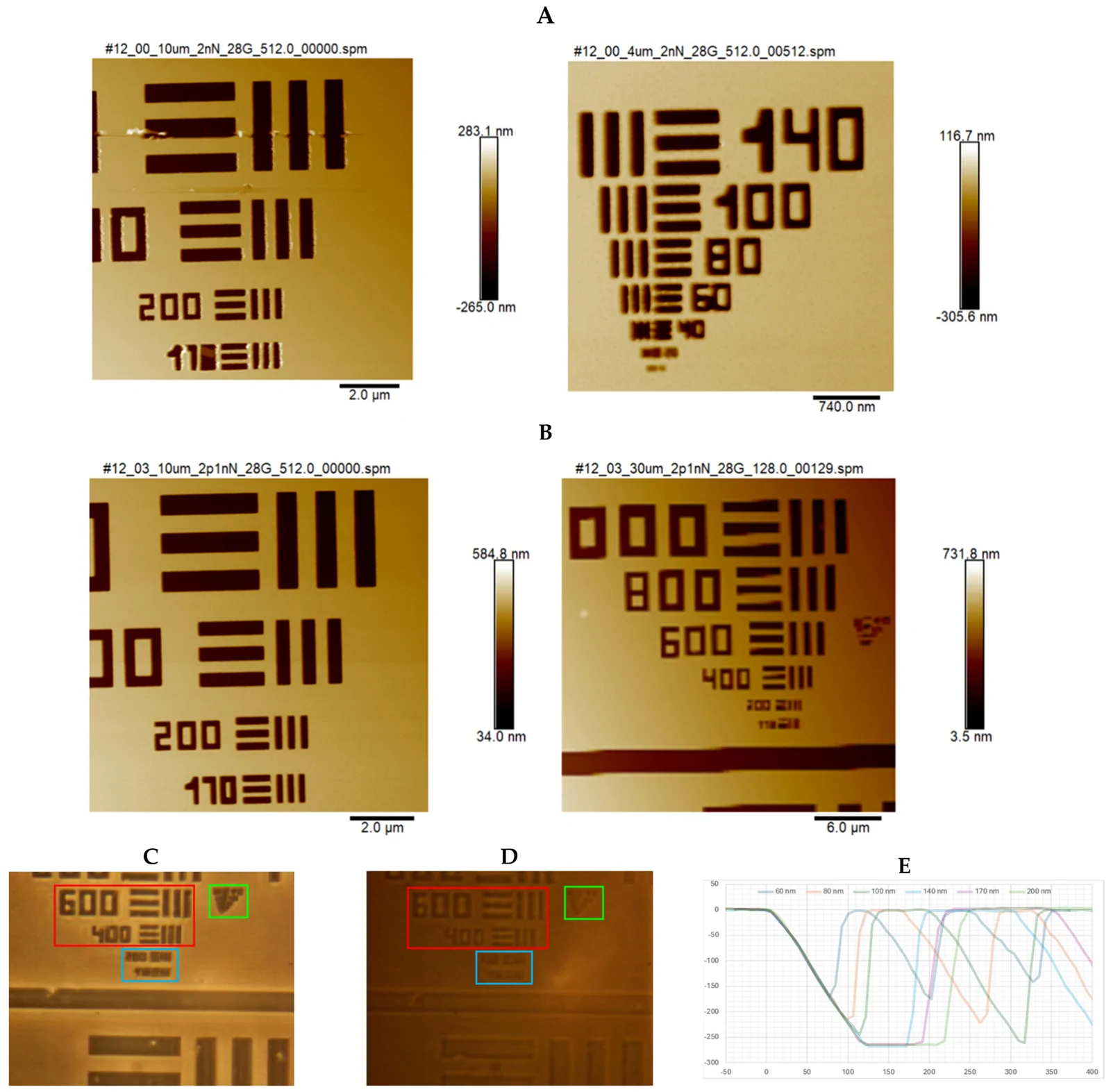
(**A**,**B**) AFM images acquired of the test target pattern fabricated in PMMA (© Jonathan D. Mar and Andrew Irvine). Selected RoIs are **#00** (**A**) and **#03** (**B**). (**C**) PCM and (**D**) DIC photomicrographs of these same regions (**#00** and **#03**, respectively) acquired under an intermediate magnification of 2.4×. The red, blue, and green boxed RoIs indicate those regions used for image SR and further analysis. (**E**) Depth map of the individual features (bars and spaces) in the test target sample, acquired via AFM. The depth profile is acquired via a single pass of the AFM probe, resulting in a sloping surface where the probe descends into the trench characteristic of the said feature. Figures are adapted from Ref. [34].

**Figure 5:**
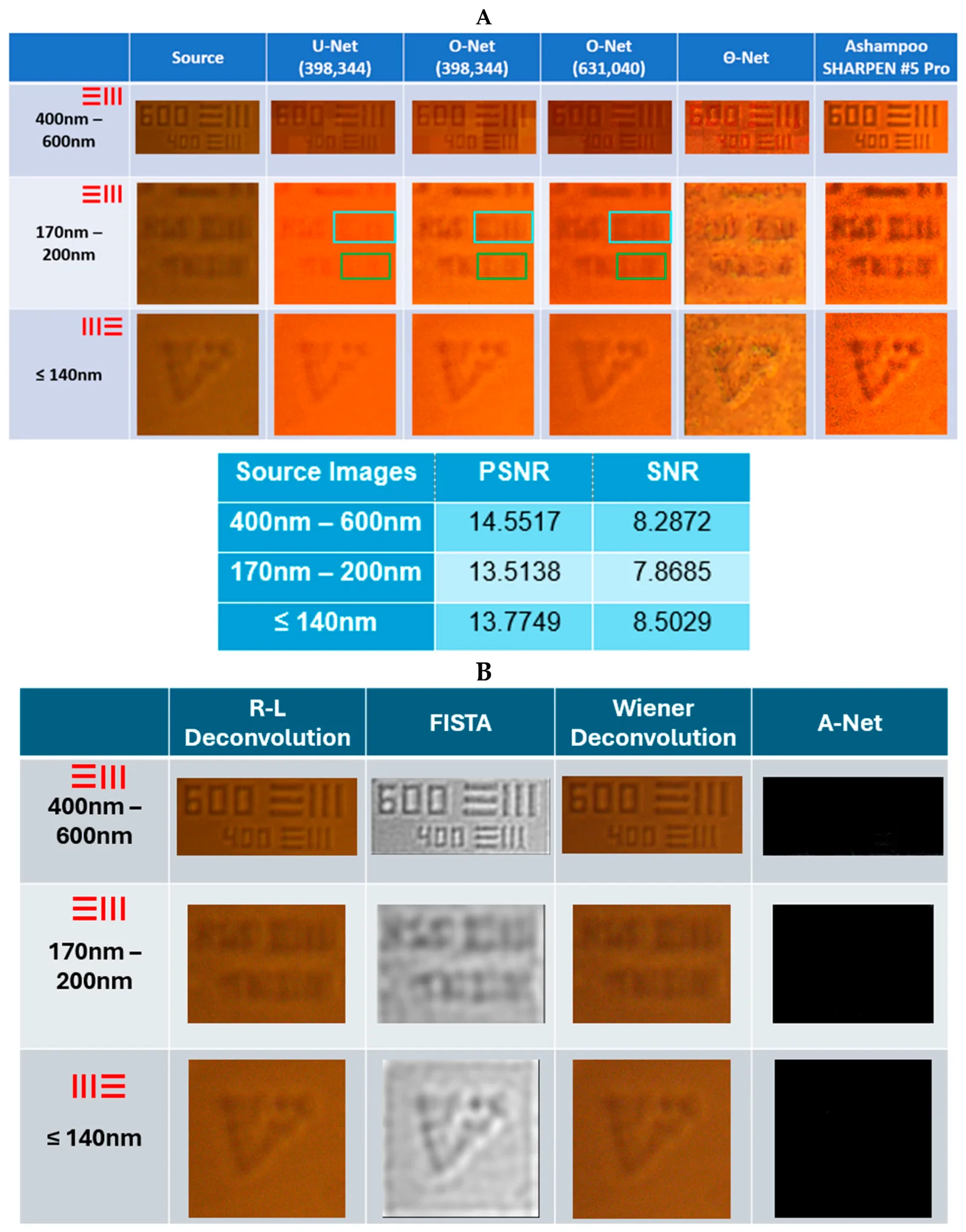
(**A**) *In silico* SR DIC micrographs of the PMMA target, employing U-Net, O-Net and Θ-Net models trained over **101 epochs** (ep) (3944 iterations/epoch × 101 epochs = 398,344 iterations) and **160 epochs** (3944 iterations/epoch × 160 epochs = 631,040 iterations) (the 101-ep models employed a 5-layer architecture while the 160-ep models used a 7-layer architecture). Here, an intermediate magnification of **2.4×** was utilised for acquisition of the raw (**Source**) images, which correspond to the individual coloured boxed RoIs shown in Figure 4D previously (red for 400–600 nm; blue for 170–200 nm, and green for ≤140 nm features). From these images, one might observe that both the 101-ep and 160-ep O-Net-generated images demonstrate a clear delineation of the darker bars from the lighter-coloured spaces between them (especially for the 200 nm bars, and perhaps less so for the 170 nm bars), facilitating resolution of the said bars (with the 101-ep O-Net model outperforming the 160-ep O-Net model), while the U-Net generated image does not show such a clear distinct boundary (as depicted by the cyan and green rectangles). Additionally, the Θ-Net-generated images depict substantial noise for features ≤ 200 nm, being far more sensitive to slight pixel variations (see Figure 5 for details). Image SNRs and PSNRs have also been included for the respective **Source** images shown in this figure. Figure is adapted from Ref. [34]. (**B**) The corresponding FoVs deconvolved using R-L deconvolution, FISTA, Wiener deconvolution and A-Net.

**Figure 6:**
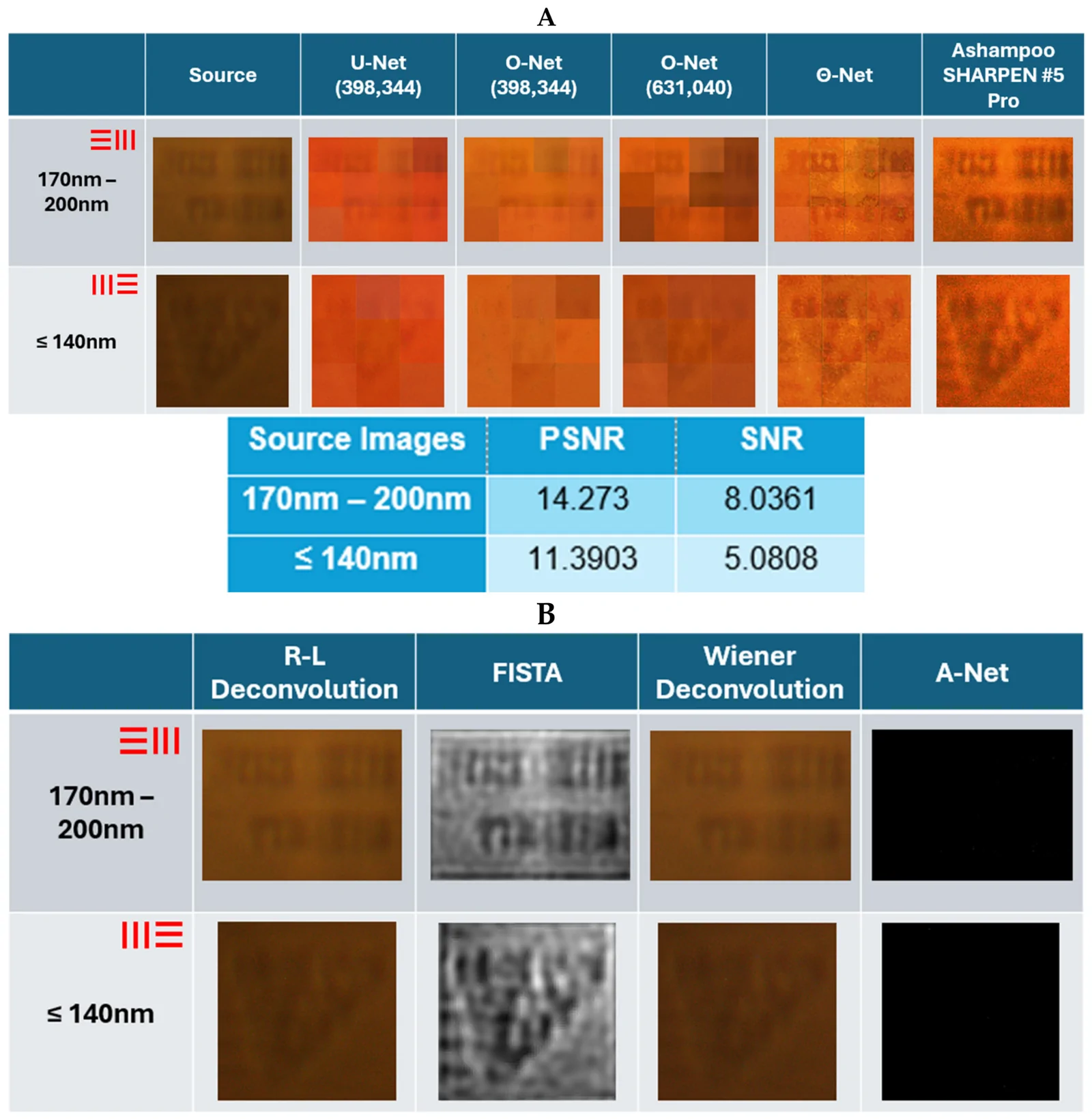
(**A**) *In silico* SR DIC micrographs of the same custom-fabricated PMMA target depicted in Figure 5 previously. Here, an intermediate magnification of **3.75×** was used instead for acquiring the raw (**Source**) images. Intriguingly, the Θ-Net models seem to generate the most well-resolved images in the current context, surpassing those of U-Net and even O-Net (for both the 101-epoch and 160-epoch O-Net models). On closer inspection, it is observed that the **Source** images have a significantly reduced contrast and SNR, making it rather challenging to resolve the individual bars in the target pattern. In such a situation, it is thus noteworthy to highlight that Θ-Net is well-placed to resolve these images, being more sensitive to minor fluctuations in pixel intensity (as mentioned previously). The SNRs/PSNRs for the **Source** images shown are generally much lower than those in Figure 5. Figure adapted from Ref. [34]. (**B**) The corresponding FoVs deconvolved using R-L deconvolution, FISTA, Wiener deconvolution and A-Net (akin to Figure 5 previously).

**Figure 7:**
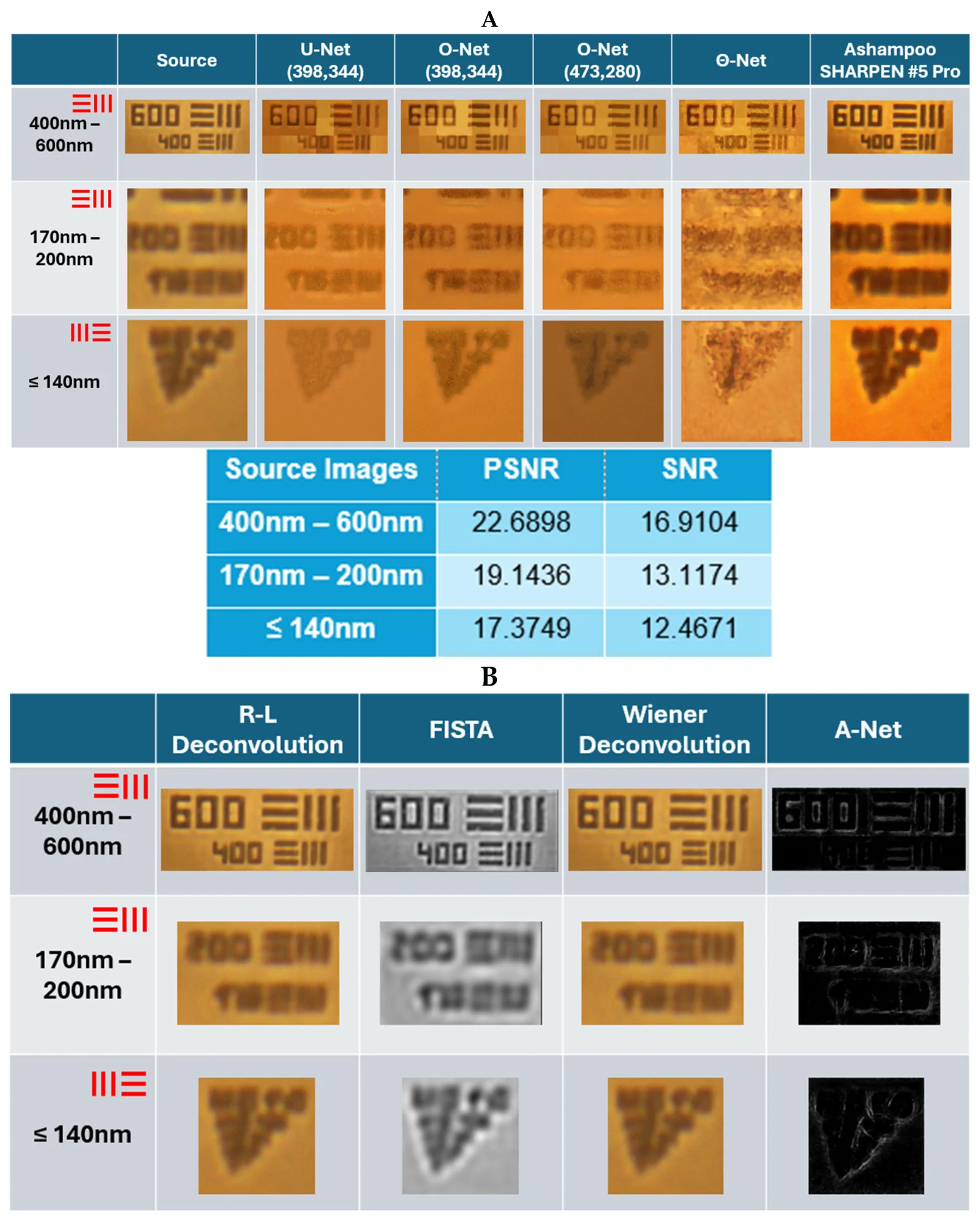
(**A**) SR PCM micrographs of the custom-fabricated PMMA target, derived *in silico* via U-Net, O-Net and Θ-Net models being trained over **101 ep** and **120 ep** (3944 iterations/epoch × 120 epochs = 473,280 iterations). As with Figure 5, a **2.4×** intermediate magnification was employed to acquire the **Source** images, with the regions cropped and tiled as described in the Section 2 (RoIs were selected as demarcated via coloured boxes in Figure 4C). Due to the relatively high SNR of these PCM **Source** images, *no* brightness/contrast enhancements were made to the generated images (unlike Figure 5 previously, where the **Source** image SNR was relatively low, requiring these enhancements to be implemented for easier visual identification, particularly for features ≤ 200 nm). Here too, both the U-Net and O-Net models produced images showing a clear separation of the bars, with the best results gleaned from the 101-ep O-Net model. The Θ-Net models, however, depict significant image noise when super-resolving the PCM images, making them unsuitable for this use case. As with Figure 5 and Figure 6 previously, the individual **Source** image SNRs/PNRs have also been tabulated in this figure. Figure adapted from Ref. [34]. (**B**) The corresponding FoVs deconvolved using R-L deconvolution, FISTA, Wiener deconvolution and A-Net.

As described in our pre-print [34], the trained models were subsequently evaluated/tested using a calibrated test-target pattern (adapted from the USAF1951 standard) and designed using KLayout 0.28.3 (© Shiraz S Kaderuppan, 2024, Singapore) which was custom-fabricated in PMMA using electron beam lithography (EBL) on a 24 mm × 24 mm square coverslip (#1.5) (© Jonathan D Mar, 2024, Toronto, Canada and Andrew Irvine, 2024, Cambridge, UK). The said target consisted of features adopting the industry-wide USAF1951 standard of equally spaced bars and spaces, with sizes ranging from 20 nm to 6 µm, thereby making it suitable for assessing the SR performance of the proposed O-Net and Θ-Net frameworks. The coverslip was then mounted onto a standard (1” × 3”) microscope glass slide using a high refractive index mountant (Norland Optical Adhesive NOA 170, n_d_ = 1.70 (at 589 nm), Norland Products Inc., Cranbury, NJ, USA), prior to image acquisition (utilising both PCM and DIC techniques) with a Leica HCX PL APO 100×/1.4 objective (Leica P/N: 506211). Different intermediate magnifications of 2.4× and 3.75× were also employed in the image acquisition, resulting in a total of 4 images (2 for each of the aforementioned modalities). When coupled with the 0.7× C-mount and intermediate tube magnification factor of 1.12× as mentioned previously, this translated to an effective overall magnification of 188.16× and 294× correspondingly, implying a Nyquist sampling limit of 50.85 cycles/μm (*s* = 9.832 nm/px) and 79.46 cycles/μm (*s* = 6.2925 nm/px) at the specimen-plane respectively (when computed using the Nyquist sampling criterion of *f*_Nyquist_ = 1/(2 *s*) [35], where *s* repents the camera pixel pitch projected onto the sample plane). Subsequently, specific RoIs were defined for the respective images, with these RoIs being subsequently cropped into individual image tiles in MATLAB R2022b (© 1984–2022, The MathWorks, Inc., Natick, MA, USA) (see Figure 4C,D for details). For the 2.4× intermediate magnification images, the RoI corresponding to the 400–600 nm features was cropped into 300 px × 300 px image tiles (total area: 2100 px × 900 px, i.e., 7 × 3 tiled matrix), while the RoI for features ≤200 nm was cropped into either 600 px × 600 px image tiles (for PCM) or 700 px × 700 px image tiles (for DIC). For the 3.75× intermediate magnification micrographs, 256 px × 256 px image tiles were cropped for the respective RoIs instead. These image tiles were then fed into the respective networks (U-Net, O-Net, Θ-Net, etc.) for generation of the SR images. Where deemed appropriate, the brightness, contrast, and sharpness of the generated images were Python-enhanced for easier visual comparison. It would be prudent to emphasise at this juncture that this does *not* affect the resolution of the image, since resolution (as defined by the Rayleigh criterion) refers to the *minimum* distance between 2 points in an image for them to be seen as separate, an aspect that refers to the level of detail present in the image (*not* its brightness/contrast or sharpness). The cropped images were then combined into a single ‘expanded view’ image where required (as depicted in Figures 5–7). Figuratively, this empirical verification process may be described by Figure 2 as follows:

To ensure proper verification of the DNN model performance (especially with regard to its potential for image SR), images of the assayed RoIs were acquired separately using AFM, where the accompanying scale bar was used for precise evaluation of the respective feature dimensions. This thus allowed comparison with the DNN-generated SR PCM/DIC images, enabling an unbiased determination of the image SR performance for each of the evaluated models. In this light, it would also be prudent to mention that the models had *not* been trained with any images of the test target sample (or even images closely resembling it) to prevent potential data leakage, which might affect the reliability of the testing process. This was further corroborated by the fact that the test target sample represents the *only* non-biological sample used in this entire study, while all of the images in the training and validation datasets were of biological origin (i.e., sections of plant and animal tissues, as well as protist samples) as described in the previous subsection. The SR PCM/DIC images were also converted to grayscale prior to the generation of an intensity profile plot along a linear portion of the image in ImageJ 1.52n (NIH, USA), so that the individual bar/space widths may be determined by a single horizontal baseline in the intensity profile plot, which establishes near-equivalent widths for these individual peaks (spaces)/troughs (bars). This approach (known as FWST) may be used to specify the minimum resolvable distance according to the Rayleigh criterion, thereby alleviating potential inaccuracies arising from simple visual observation and determination of the minimum resolvable distance. A similar method employing profile lines drawn across specific features for elucidation of the image resolution has also been described in Ref. [36]. High levels of noise present in the grayscale images (preventing the direct determination of the respective bars and spaces from the intensity profile plot) necessitated the corresponding distances to be inferred by comparing the SR PCM/DIC image against the respective AFM image, when extrapolated onto the intensity profile plot (see Figures 9 and 10 for details). The AFM-calibrated test target thus provides an independent physical reference for the nominal feature dimensions, while the reconstructed-image measurements are further evaluated *quantitatively* using intensity profiles and complementary image quality assessment (IQA) metrics, which are discussed in the subsequent sections.

### 2.4. Comparison of Model Performance Against Other Widely Utilised Protocols for Image SR

Separately, the acquired images were also processed using Richardson-Lucy (R-L) deconvolution, Wiener deconvolution, Fast Iterative Shrinkage Thresholding Algorithm (FISTA) and A-Net (a DNN architecture for SR fluorescence nanoscopy described in Ref. [17]). These methods represent established computational image-restoration and super-resolution approaches that were selected as comparative benchmarks for O-Net and Θ-Net under the parameterisations described in Table 2 as follows:

R-L and Wiener deconvolution were applied to the input (**Source**) images using MATLAB R2022b (© 1984–2022, The MathWorks Inc., Natick, MA, USA), while FISTA was performed in DeconvolutionLab2 2.1.2 (© 2010–2018, Biomedical Imaging Group, EPFL). A-Net was downloaded from Ref. [37] and installed as a plug-in to ImageJ, and subsequently deployed on the **Source** images using the pre-trained model for tubulin [as this was the closest match to the linear features (bars) on the sample]. However, as these pre-trained models only accepted images with dimensions of 512 px × 512 px, successful implementation of A-Net in this context required the **Source** images to first be converted to grayscale and subsequently cropped into 512 px × 512 px image tiles (in MATLAB R2022b).

### 2.5. Quantitative Evaluation of Model Performance and SNR Elucidation

In the present study, the signal–noise ratio (SNR) and peak SNR (PSNR) of the **Source** images were utilised as quantitative metrics for benchmarking model performance. The respective formulae (for SNR and PSNR) may be described by Equations (2) and (3) below (from Ref. [21]):

(2) $$\mathrm{SNR}=10\;\mathrm{lg}\left(\frac{S}{N}\right)$$

(3) $$\mathrm{PSNR}=10\;\mathrm{lg}\left(\frac{{\mathrm{PV}}^{2}}{\mathrm{MSE}}\right)$$

where S denotes the signal, N represents noise (in volts), PV is the maximum value of a pixel in an image (i.e., 255 for an 8-bit image), and MSE refers to the mean squared error [21]. Here, the SNR and PSNR values may be computed in MATLAB R2022b using the built-in PSNR MATLAB function, implying the pixel-level definitions for S, N, PV and MSE are also adapted from MATLAB in this respect. Notably, determining the SNR/PSNR required the **Source** images to be compared against their corresponding *reference* images, which were obtained through a deconvolution protocol applied to the respective **Source** images within Ashampoo SHARPEN #5 Professional (64-bit) v5.41.04116 (Accelerated Vision UG, Kiel, Germany).

In addition to the above metrics, four other quantitative IQA approaches [38] were also utilised in the present study. These were the *contrast–noise ratio* (CNR), *contrast transfer function* (CTF), *correlation mean* (between the raw intensity profile plot and the square wave) and *full-width-at-half-maximum* (FWHM). The formulas and experimental procedures employed for the determination of each of these metrics are detailed in the following paragraphs.

CNR and CTF may be described by Equations (4) and (5) as follows:

(4) $$\mathrm{CNR}=\left|\frac{<S>-<B>}{\sigma_{N}}\right|$$

(5) $$\mathrm{CTF}=\frac{I_{\max}(f)-I_{\min}(f)}{I_{\max}(f)+I_{\min}(f)}$$

where <*S*> denotes the mean *signal* intensity (e.g., of a bar), <*B*> represents the mean intensity of the *background* (i.e., spaces), and *σ_N_* represents the standard deviation of noise within the RoI. *I_max_*(*f*) and *I_min_*(*f*) refer to the maximum and minimum intensity of a periodic test pattern at spatial frequency *f* in the raw intensity profile plot respectively (the interested reader is hereby encouraged to refer to Refs. [39,40] for further details on the elucidation of each of these IQA metrics).

At this juncture, it would be useful to mention that a square wave profile is mapped onto the raw intensity profile plot across 5 linear profiles (per RoI) for determination of these IQA metrics. A square wave profile is employed in the current context as the target contains discrete horizontal and vertical *bars* rather than *points* (which would correlate to a sinusoidal input); hence, the resulting measurement may be more appropriately regarded as CTF, instead of MTF (*modulation transfer function*). Furthermore, the relationship between a square-wave response and a sinusoidal response had also been previously established by Ref. [41], which showed that the sinusoidal response can, under appropriate linear-system assumptions, be reconstructed from square-wave responses through the corresponding harmonic series [41], an example of which may be utilised in traditional spectral deconvolution protocols (such as Fourier decomposition, amongst others). In this respect, CTF was retained as the primary direct measurement from the bar target in the present analysis, rather than being equated to MTF. Separately, CNR was also included in this study as it would demonstrate the model’s ability to effectively differentiate between the signal, background and noise pixels; a higher CNR being indicative of image (*contrast*) enhancements applied to the feature-based pixels (*signal*), while the background contrast and image noise are reduced. At this juncture, it would be prudent to emphasise that this *selective* contrast enhancement is substantially different from a *holistic* enhancement of the overall image brightness/contrast/sharpness using Python (as indicated in Figure 2 previously); the latter being useful for improving visual clarity, ***not*** image resolution (since it is based on raw pixel intensity values that do not distinguish between features and background/noise).

**Figure 3:**
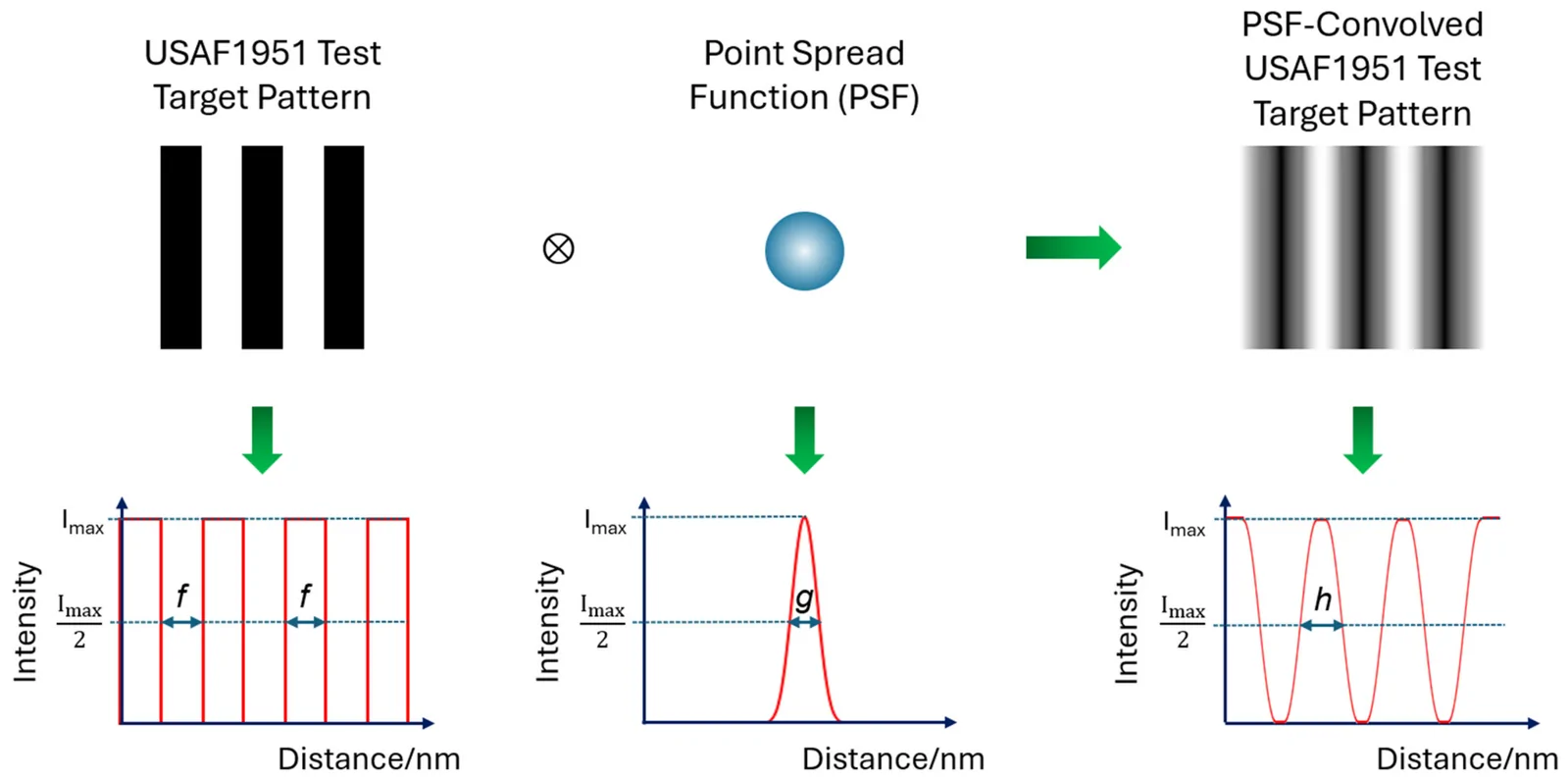
Expected intensity profile plots depicting the convolution of the test target image with the PSF of the optical train. Notice the resultant square sinusoidal wave, which may be approximated either by a square wave or a sinusoidal function, although the former is closer to the unconvolved impulse response of the sample (ground truth) and hence is utilised in the present study. *f, g, h*: full width at half maximum (FWHM); ⨂: 2D convolution.

In addition to CNR and CTF, the correlation mean and FWHM of the square wave plot were also determined in the present study for each of the assayed line profiles—the correlation mean describes the average correlation between the raw intensity profile and the square wave mapped onto it on a pixelwise basis, while the FWHM (which often relates to the width of a wave profile at its mid-amplitude) may, for uniform square waves (as expounded in the current context), also be translated to closely approximate the feature width (please see Figure 3 for details). Computed FWHM values <25% of the nominal feature width were discarded, as this would indicate noisy pixels being translated into discrete waves on the raw intensity profile plot. This facilitated the derivation of ΔFWHM, which describes the absolute difference between the FWHM (an empirically determined quantity obtained from the raw intensity profile plot) and the nominal stated width of the feature in question. As such, a smaller ΔFWHM would imply a closer correlation between the empirically measured mid-amplitude width of the square wave profile and the nominally stated width of the assessed feature (e.g., 600 nm, 400 nm, etc.), demonstrating the model’s ability to super-resolve the input images—an aspect that may be further verified through the derived *correlation mean* in the present context.

### 2.6. Acquisition of Emission Spectra

Separately, a spectrophotometer (Vernier Instruments SpectroVis Plus ^®^, Beaverton, OR, USA) was also used to obtain the emission spectra of the halogen light source at different intensity values. These emission spectra were used for determining the peak emission wavelengths (λ_peak-em_) of the light source, which may then be employed for the computation of the theoretical resolution attainable through the utilisation of the full numerical aperture (NA) of the objective lens [using the Rayleigh diffraction criterion as described by Equation (1) previously]. Notably, as a broadband halogen illumination source is being employed in the present study, the peak value of λ_em_ was determined for the *yellow-orange* region of the emission spectrum [as this correlated visually with the observed field-of-view (FoV)], with this value being subsequently used to calculate the lateral resolution of the optical train under the established hyperparameters. In addition, $\bar{\lambda_{\mathrm{em}}}$ was also determined as a *weighted average* of the visible spectrum wavelengths (ranging from 399.9 nm to 700.5 nm) based on their relative spectral emission intensities as described by Equation (6) below (with $\bar{\lambda_{\mathrm{em}}}$ also being used for computing the Rayleigh lateral resolution in the current context):

(6) $$\bar{\lambda_{\mathrm{em}}}=\;\frac{\sum I\lambda }{\sum I}$$

where I represents the relative intensity of the measured wavelength λ in the emission spectrum.

From this determination, we may subsequently be able to identify the additional resolution enhancement (if any) provided by utilising the aforementioned O-Net and Θ-Net models.

## 3. Results

The U-Net, O-Net and Θ-Net models, as described in the previous Section 2, were assessed on their image SR generation performance using the calibrated test-target slide, and the results of these analyses are presented in Figure 4 [34]:

### 3.1. AFM Image of the Test Target Sample

Analysis of the emission spectrum of the halogen light source revealed its peak (non-red) excitation wavelength to be 619.4 nm. Applying the Rayleigh diffraction-limited formula [Equation (1)] for the computation of lateral resolution d_xy_, we arrive at a value of **329 nm** for d_xy_ (when λ = 619.4 nm) (see Supplementary Figure S2 for details). Nonetheless, it would be imperative to highlight here that the computed 329 nm value represents a nominal reference diffraction limit calculated using the measured peak wavelength, rather than a complete broadband optical-resolution characterisation. Separately, we have also considered adapting the approach of *multiwavelength weighting* as prescribed in Ref. [42], where a weighted average is obtained of the different measured wavelengths with regard to their spectral emission intensities. Doing this resulted in an effective wavelength of **654.7 nm** when the entire measured emission spectrum was used (corresponding to wavelength values ranging from 380 nm to 899.6 nm), or **612.2 nm** when only the visible portion of the emission spectrum was considered (ranging from 399.9 nm to 700.5 nm). Between these two values, a wavelength of 612.2 nm was closer to the observed yellow-orange colour of the halogen lamp emission (654.7 nm was in the red region, and hence was not reflective of the actual visual observation noted from the FoV of the currently acquired images). Inputting this value of 612.2 nm into the Rayleigh formula results in an effective resolution of **325 nm**, which is very close to the aforementioned 329 nm diffraction limit. Despite this, it would be prudent to emphasise that the actual effective resolution of the microscope is influenced by the spectral bandwidth of the illumination, objective transmission, detector response, condenser NA and other components of the optical transfer function. Accordingly, the reported 325/329 nm values are now employed as a theoretical reference against which the computational reconstruction is compared, rather than as exact empirically determined resolution limits.

**Figure 8:**
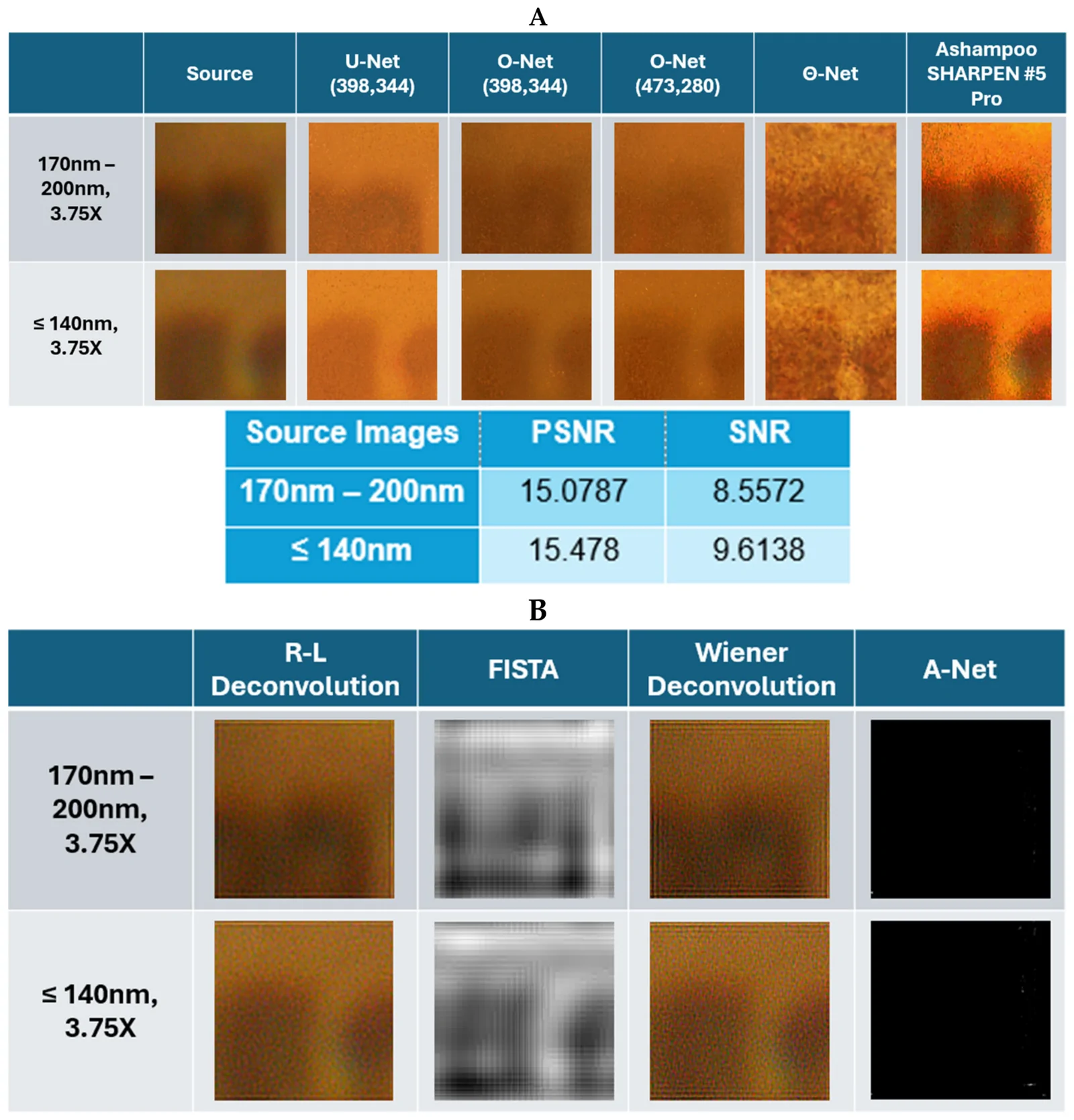
(**A**) DNN-facilitated SR PCM micrographs of an image tile of the custom-fabricated PMMA target, employing U-Net, O-Net and Θ-Net models trained either over **101** or **120 epochs**. As with Figure 6 previously, a **3.75×** intermediate magnification was used in capturing the raw (**Source**) images for this figure. Also, as mentioned in the Figure 6 caption, the Θ-Net models have once again performed extremely well under this intermediate magnification, transcending both the U-Net and O-Net models when the **Source** image has a very low SNR (as depicted in the table), comparable to that of Figure 6. Figure adapted from Ref. [34]. (**B**) The corresponding FoVs deconvolved using R-L deconvolution, FISTA, Wiener deconvolution and A-Net.

**Figure 9:**
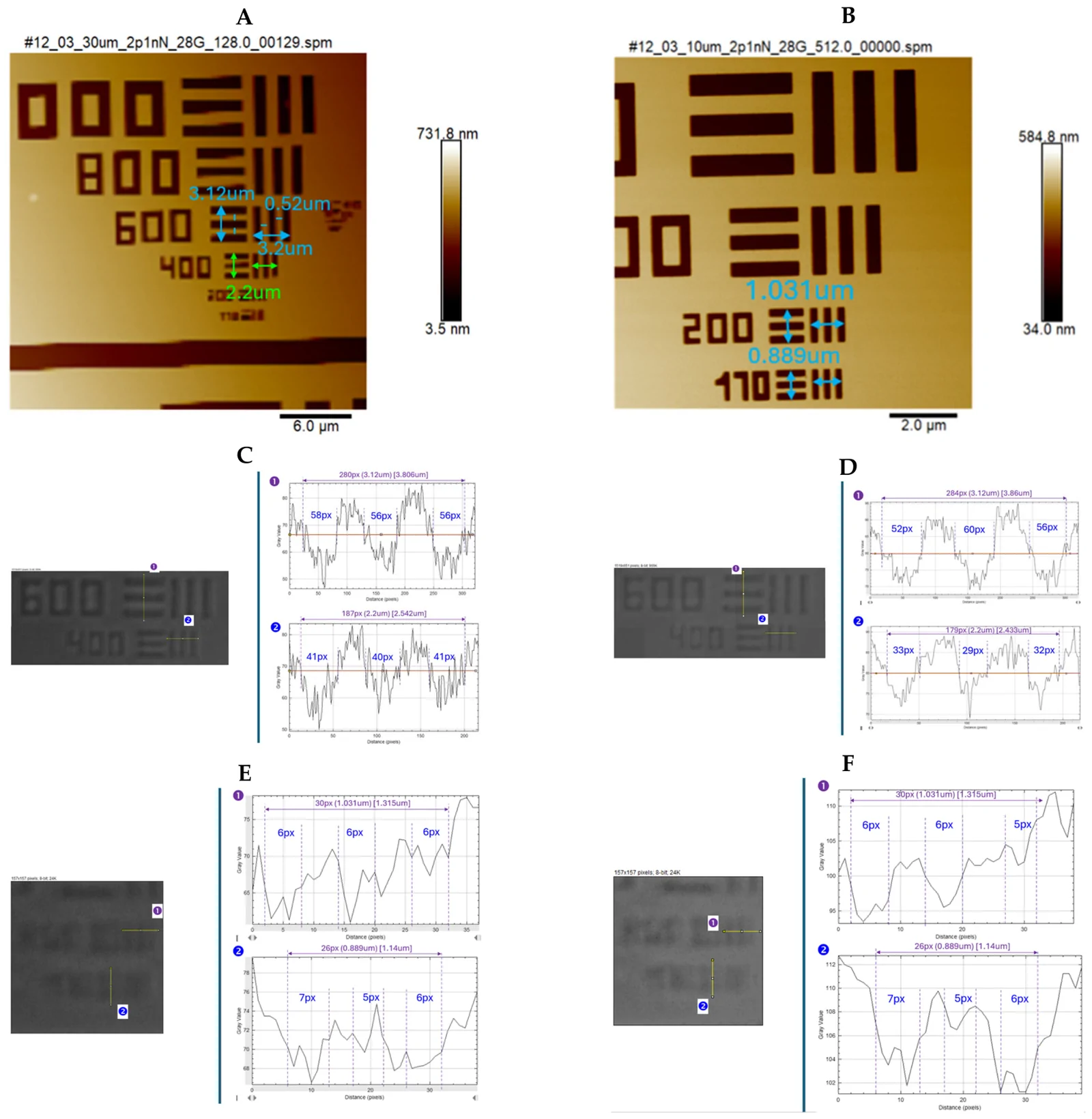
(**A**,**B**) AFM-calibrated micrographs with their bar and space widths measured and indicated using the associated scale, with their respective grayscale-converted *in silico* SR DIC images (generated by the 101-ep O-Net model portrayed in Figure 5 previously) and intensity profile plots (**D**,**F**), as well as the corresponding **Source** images (**C**,**E**). In panels (**C**–**F**), the measurements derived from panels (**A**,**B**) are indicated in parentheses, while the values in square brackets indicate the computed (*theoretical*) values as derived from the compounded magnification of the microscope’s optical train. From the intensity profile plots in panel (**F**), elevated levels of image noise (i.e., reduced SNR) make it highly challenging to isolate signal from noise using just a single linear threshold (as might commonly be done in image analysis). Due to this, separating the features (bars and spaces) is primarily achieved through visual comparison with the AFM micrograph (panel (**B**), associating the fluctuations in pixel intensity with the actual grayscale values in the SR DIC images (panels (**D**,**F**)). Using our previous determination of the native diffraction-limited resolution as 329 nm (or the weighted average value of 325 nm), one may realise that the features in panel (**F**) are well below the diffraction limit. Panels (**C**,**D**), however, do not depict these issues since their features are above the diffraction limit and are easily distinguished, allowing a single threshold (brown line) for feature isolation. Figure source: Ref. [34].

**Figure 10:**
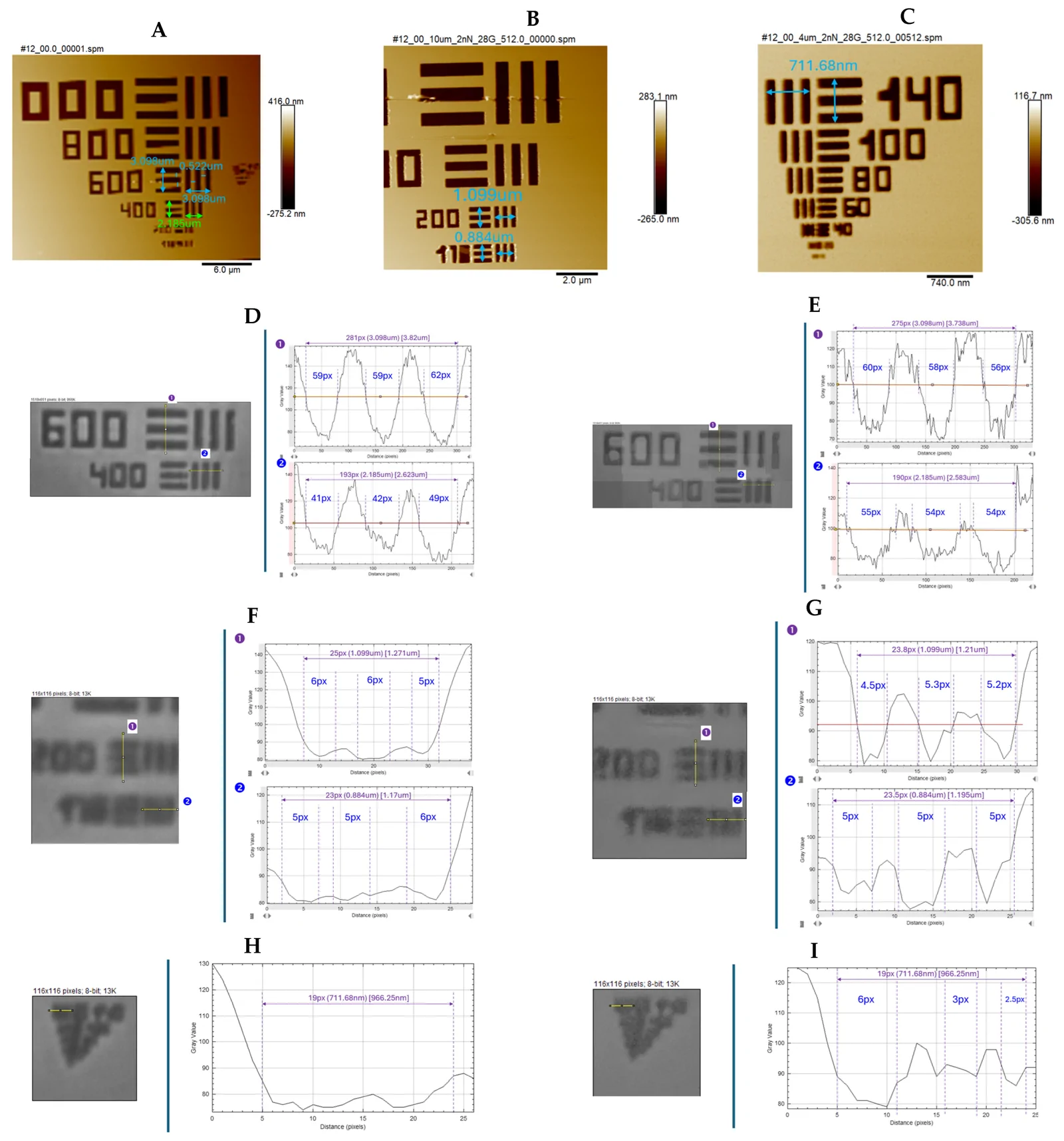
AFM-calibrated images with their respective measurements (panels (**A**–**C**) coupled with their respective grayscale *in silico* SR PCM micrographs with the corresponding intensity profile plots generated via the 101-ep O-Net model (as shown in Figure 7) (panels (**E**,**G**,**I**)) and their input **Source** images (panels (**D**,**F**,**H**)). As in Figure 9 previously, the values in panels (**D**–**I**) indicate the measurements derived from panels (**A**,**B**) in parentheses, while the values in square brackets denote the theoretical values computed from the overall magnification of the microscope’s optical train coupled with the camera pixel size. Notably, the discrepancies between the theoretical and empirical values may be attributed to the fuzzy edges of the bars in the test target pattern, making it difficult to accurately define the boundary between a bar (trough) and its adjacent space (peak); an issue that also resonates with Figure 9. For this reason, the theoretical values are not utilised in computing the resolution of the image. Also, higher levels of image noise in the SR PCM micrograph in panel (**I**) (for the ≤140 nm features) make it difficult to discriminate signal from noise; hence, a similar approach adopted for Figure 9 was also utilised in this context (i.e., the AFM micrographs were compared against the corresponding grayscale images to elucidate the boundaries segregating the bars from the spaces). The features in panel (**H**), however, could not be resolved; an indication that these features lay well below the resolution limit. The 200 nm features in panel (**G**) were easier to resolve (attributable to the higher SNR of this RoI), whereas the 170 nm features depicted increased image noise (similar to panel (**I**), making it more challenging to isolate individual bars and spaces (a similar trend was also noted for panel (**F**), although the features were not as clearly discernible as those in (**G**), both visually and as evident from the intensity profile plot). As for panels (**D**,**E**), the intensity profile plots do not manifest such issues, since their features are generally larger than 329 nm (or the weighted average value of 325 nm); hence, a single linear threshold may be used to demarcate the features (as in Figure 9). Additionally, the 200 nm (and 170 nm) features in panel (**G**) may also be considered to be far smaller than the diffraction limit (325/329 nm) despite being clearly visible in the O-Net model-generated image, demonstrating the capability of these models for SR PCM in the current context. Figures are adapted from Ref. [34].

The test target slide was then imaged via PCM and DIC microscopies (described in Section 2 previously) and subjected to *in silico* image SR via application of the aforementioned models. Figure 5, Figure 6, Figure 7, Figure 8, Figure 9, Figure 10, Figure 11 depict the results of this analysis (adapted from Ref. [34]).

### 3.2. DIC/SR DIC Image of the Test Target Sample

### 3.3. PCM/SR PCM Image of the Test Target Sample

Subsequently, the **Source** images for each of the boxed RoIs (depicted in Figure 5 and Figure 7) were converted into grayscale in ImageJ 1.52n, prior to the intensity profile being plotted for a linear RoI defined in the image (as described in the Section 2). The results of this analysis are depicted in Figure 9 and Figure 10 as follows:

### 3.4. Quantitative Intensity Profile Analysis of the SR DIC and SR PCM Micrographs

### 3.5. Quantitative Analysis of SR DIC and SR PCM Images via Aforementioned IQA Metrics

**Figure 11:**
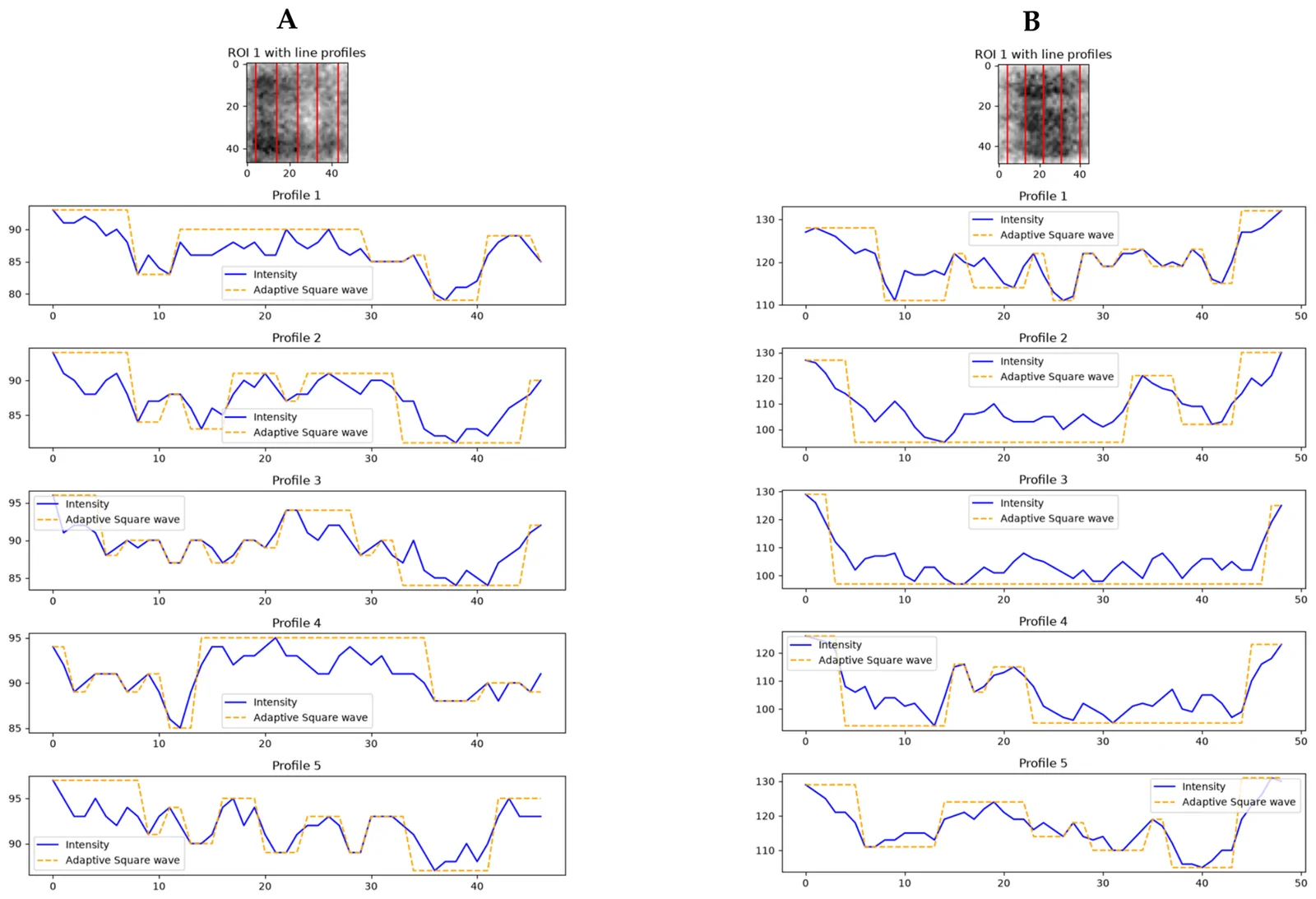

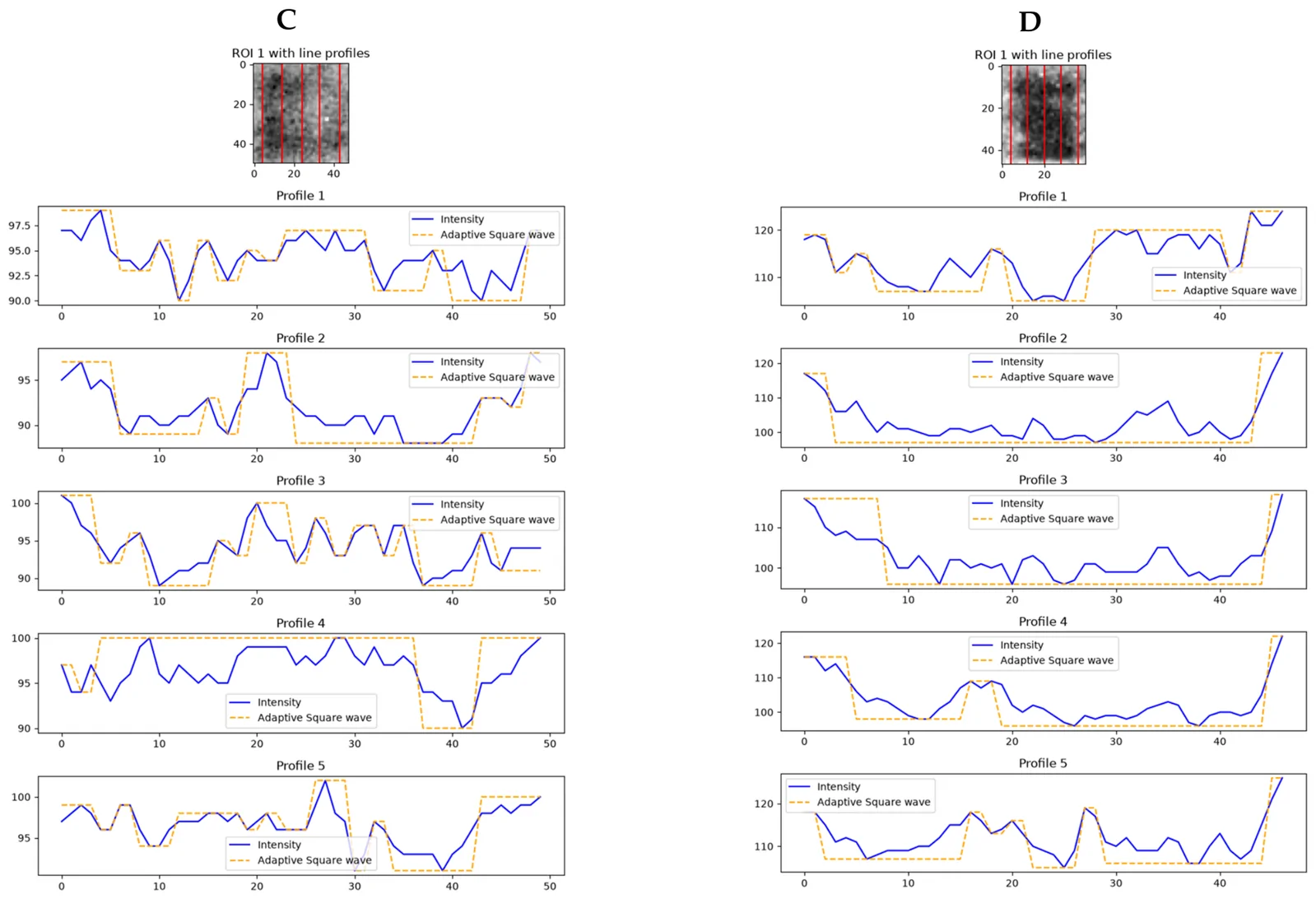

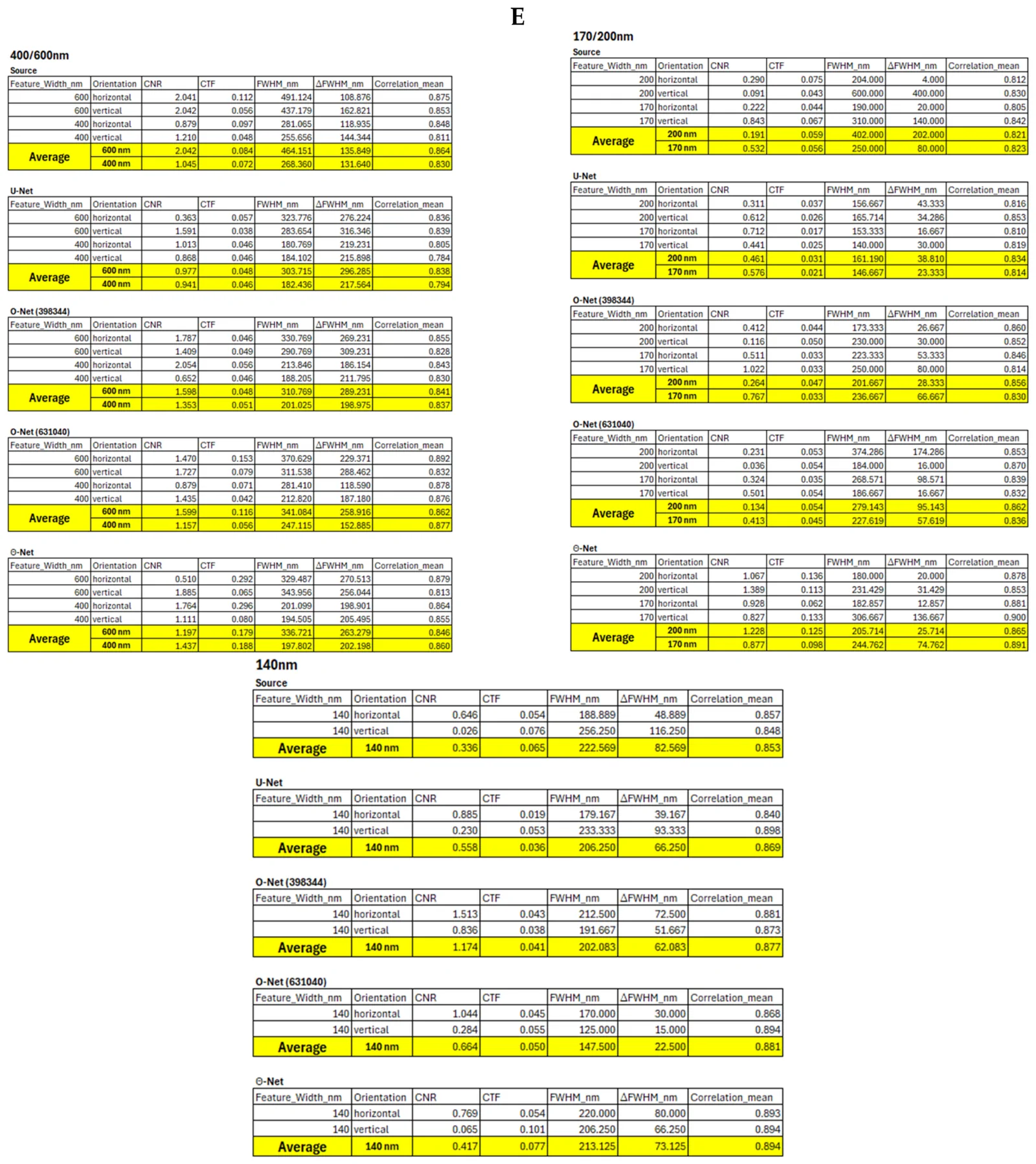

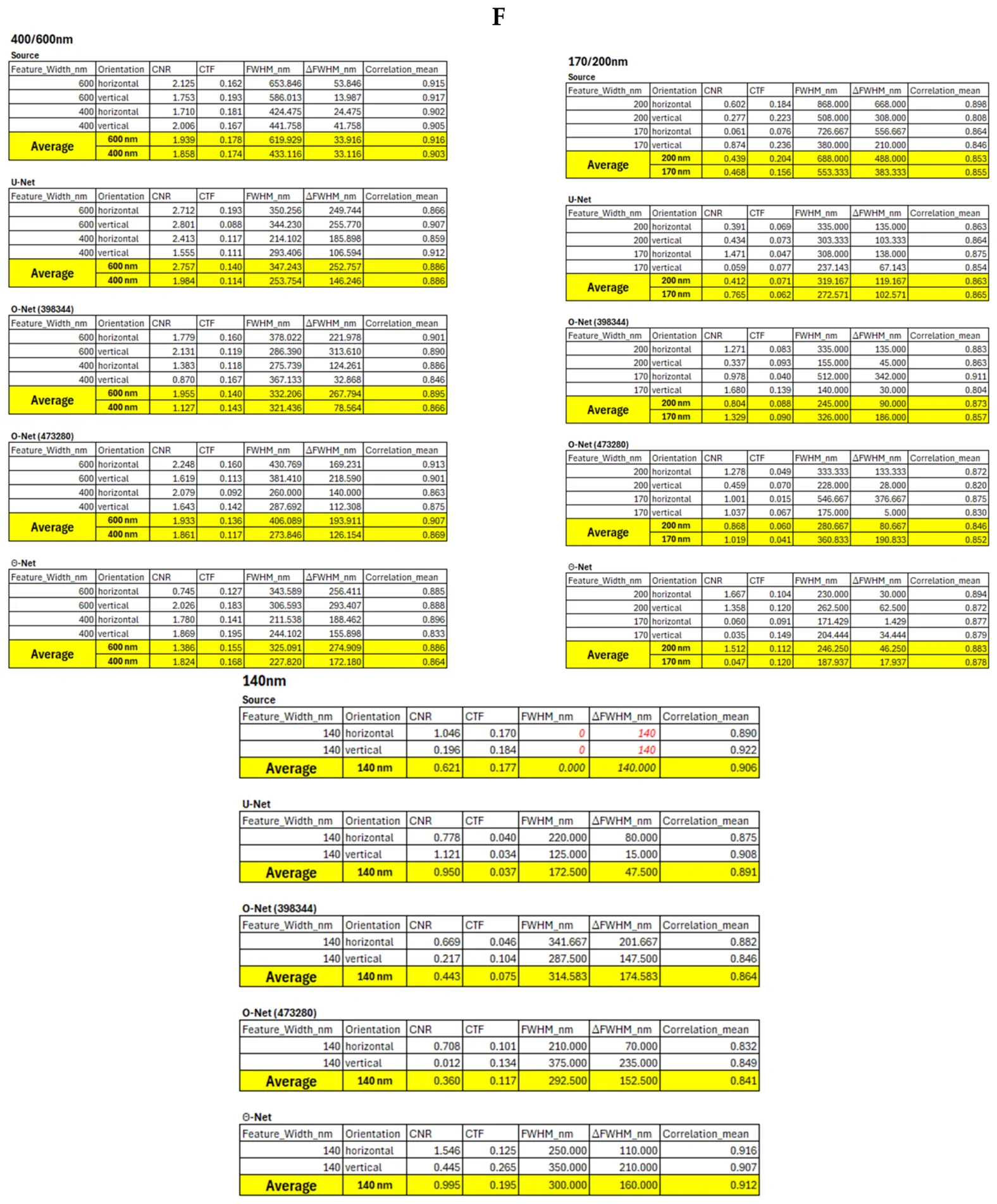
Raw intensity profile plots for SR DIC (**A**) and SR PCM (**B**) depicting the O-Net model-generated images for 200 nm horizontal features (*bars*), coupled with their corresponding mapped square waves. A similar pair of raw intensity profile plots for the U-Net model-generated images of the same 200 nm horizontal bars is also shown for SR DIC (**C**) and SR PCM (**D**) respectively. (**E**,**F**) show IQA metric values for the SR DIC (**E**) and SR PCM (**F**) images assayed in the present study, with nominal feature sizes (in nm) in the range of [140,170,200,400,600] respectively. Crucially, it may also be noted that the 140 nm features in the SR PCM **Source** images do not exhibit a numerical FWHM, as the bars and spaces appear to be blended together (see Figure 10H for details), resulting in the absence of a visibly distinct boundary to effectively define the FWHM in the present context.

## 4. Discussion

From Figure 5 and Figure 7, it may be observed that the 101-epoch (101-ep) O-Net models perform well in generating SR DIC and PCM images when image noise levels are low (i.e., high SNR), to the point of surpassing U-Net and Θ-Net models when paired alongside their corresponding AFM images. Notably, the Θ-Net models produce images with high levels of image noise and artifacts, portraying the Θ-Net model architecture’s sensitivity to slight fluctuations in pixel intensity (triggering the formation of artifacts fuelled by noise). However, the converse is observed when evaluating images with a *reduced* SNR (i.e., high noise levels), such as is present under a higher intermediate magnification (3.75×), where the Θ-Net models have been reported to outperform both the U-Net and O-Net models in their generative SR potential instead (see Figure 6 and Figure 8 for details). A comparison drawn between input image SNRs for the performance of the O-Net and Θ-Net models reveals a putative threshold SNR of ~7.5 (for DIC) and ~11 (for PCM), i.e., input (**Source**) image SNRs below these thresholds would favour the application of Θ-Net models (while the converse of higher SNRs would incline towards O-Net models) for image SR in phase-dependent optical microscopy. Nonetheless, it would be prudent to emphasise here that these values should be regarded as *empirical*, dataset-specific SNR ranges associated with the observed change in relative O-Net/Θ-Net performance, rather than as definitive, ubiquitously established thresholds.

At this juncture, it would be prudent to highlight the potential emergence of network hallucinations in the model-generated images, particularly for low SNR images. Here, the O-Net and Θ-Net model architectures are designed to counter possible network hallucination artifacts through a couple of important design considerations as follows:

(i) Performing a transposed convolution operation on the **Source** (input) images before convolution. Transposed convolution is an upsampling operation that adds features into a sample space, while convolution represents the reverse (i.e., a downsampling operation that reduces the feature space). By doing transposed convolution before convolution, spurious/random features added into the sample space by the model’s transposed convolution layers during training will be removed from the subsequent layers during convolution. This ensures that the final output of the model approaches the **expected** (ground truth) images while minimising potential hallucination artifacts.
(ii) Removal of a pooling layer (e.g., *max pooling*, which is common in U-Net). Pooling operations often retain key learnt features (which have the greatest impact on the output) while discarding features that are regarded as less essential (to reduce network bloat and increase prediction efficiency by addressing overfitting). However, as most nanoscale features would belong to the latter, pooling might undesirably remove these features, preventing potential image SR from being achieved in this context.

Another key point to be highlighted here refers to how the SR PCM O-Net models seemingly resolved features down to 170 nm (depicted in Figure 10G) while the SR DIC O-Net models were capable of resolving features down to 200 nm (Figure 9F), thereby surpassing the theoretical Rayleigh diffraction limit of 325/329 nm (see Supplementary Figure S2 for details) and demonstrating the capability of these DNN models for use in label-free computational super-resolution applications, particularly in extending the spatial information recoverable from conventional phase-dependent microscopy images. PCM (as a microimaging modality) generally results in images depicting an *enhanced* contrast (unlike DIC), allowing even structures below the diffraction limit to be visually discernible (despite not being resolvable). Consequently, visual detectability of a sub-diffraction feature in a PCM image should not (by itself) be taken as conclusive evidence of optical super-resolution. The resolution assessment in the present study therefore relies on *quantitative* analysis of the intensity profile plots (as described in Figure 9 and Figure 10), rather than visual identification of individual features alone (a common practice adopted in the industry). Nonetheless, it would be essential to highlight here that the underlying optical transfer function (OTF) of the microscope has not been physically altered; hence, any claims to image SR by the models in the present context would likely have to be regarded as **computational super-resolution/reconstruction**, rather than direct PSF-based SR.

At this juncture, it would also be viable to address the reasons underlying the choice of a *single* wavelength (specifically 619.4 nm or 612.2 nm) for substitution into the Rayleigh formula to compute the diffraction limit of 329 nm (or 325 nm), instead of computing the mean wavelength across the spectrum based on the relative intensities of each component wavelength. In the present context, a halogen lamp is used to illuminate the specimen, while the Rayleigh formula only accepts a *single* wavelength value (*not* a range of values) for elucidating the optical system resolution. Conventionally, this would be regarded as the mid-spectrum wavelength of the visible light spectrum (i.e., λ = 550 nm) for resolution computation using a broadband illumination source (such as the aforementioned halogen lamp), since this represents the average wavelength in the visible light spectrum range (as described in Ref. [43]). This is in contrast to the use of a *monochromatic* illumination source (such as a laser), which (employing a single illumination wavelength) would be unambiguous for resolution determination purposes. However, 550 nm actually corresponds to *green* light, *not* white (or the characteristic yellow-orange) light often observed in the emission spectrum of a halogen lamp. As such, it would be more viable to determine the *peak* wavelength in the *yellow-orange* region of the halogen emission spectrum instead, and use this value to compute the attainable resolution of the microscope’s optical train. The weighted mean of *all* the measured wavelengths is also not considered in the present context, as it suggests a red-shifted computed value of **654.7 nm** (due to the higher proportion of red and infrared light in the halogen emission spectra), which is not representative of the observed FoV. However, if a weighted mean of only the visible light wavelengths (ranging from 399.9 nm to 700.5 nm) is considered, a value of **612.2 nm** is obtained, which also corresponds to the *yellow-orange* region of the halogen emission spectrum. Through this approach, we arrive at a theoretical resolution limit of either **325 nm** or **329 nm** for the current optical setup—a finding that also seems to be supported *empirically* by the acquired PCM and DIC micrographs (as depicted in Figure 4C and Figure 4D respectively), where the 200 nm features are not clearly resolved due to them being below the calculated resolution limit. In this light, the subsequent recovery of 170–200 nm features following DNN-based reconstruction should therefore be interpreted in the context of *computational super-resolution*, rather than as a direct alteration of the diffraction-limited response of the microscope’s optical train.

On a separate note (and as indicated previously), the level of noise present in the **Source** images plays a fundamental role in influencing the visual/perceptible quality of the generated SR output images for each of the models assayed in the present study—a finding that corroborates that found in prior work exploring the use of other DNN models for fluorescence SR microscopy (such as Refs. [31,32]). Nonetheless, it would be prudent to emphasise here that although there is *no* definitive way to ascertain the exact nature of the noise in the dataset, we strongly suspect that it is comprised of multiple types of noise, including Poisson and/or periodic noise types (a detailed review on the various types of noise being described in Ref. [44]). Both Poisson and periodic noise may be deduced to be present, as the SR DIC **Source** image tiles for the features ≤ 200 nm (Figure 5A) depict seemingly imperceptible differences between the background and the features (*bars*), although this noise becomes somewhat apparent in the Θ-Net model-generated images (where the background and edges of the features become considerably grainy). This also concurs with the nature of these two noise types (as discussed in Ref. [44]), since periodic noise is “often correlated with the image content, making it difficult to distinguish from the underlying image structure”, while Poisson noise is commonly encountered under low-light imaging conditions reminiscent of the present context, where the use of a high overall optical magnification results in a reduced intensity (Intensity ∝ Magnification^−2^) [45]. In addition, small amounts of Gaussian noise may also be present in the SR DIC **Source** image tiles for the 400–600 nm features (Figure 5A). Nonetheless, removing this noise using traditional image denoising filters (including Fourier-based methods, wavelet denoising, etc.) may risk the removal of some of the nanoscale structures as well (i.e., *pseudo* noise, as highlighted in one of our previously published articles at Ref. [21]), impeding the successful recovery of these nanoscale features from the input (**Source**) images. For this reason, the use of such denoising approaches is not prescribed in the current study, where the recovery of nanoscale structures/features is essential.

### 4.1. Quantitative Analysis of the SR DIC and SR PCM Images via the Aforementioned IQA Metrics

The IQA metrics employed in this study (CNR, CTF, FWHM, and mean correlation) were selected to provide complementary measures of image quality and to assess different aspects of the agreement between the reconstructed intensity profiles and the corresponding theoretical reference profiles. As described previously in the Section 2, square-wave profiles were adopted as the theoretical reference, since the regular bars and spaces of the USAF1951 test target pattern are expected to produce alternating regions of approximately uniform intensity. In an actual imaging system, these profiles may be modified by convolution with the system PSF, resulting in a smoothed waveform that reflects the spatial-frequency response of the optical system and its associated impulse response (see Figure 3 for details). Comparison of the experimentally obtained intensity profiles with the corresponding square-wave references thereby provides a reliable means of assessing the fidelity with which the reconstructed images reproduce the nominal test-target structure.

Figure 11A–D present representative intensity profiles obtained along the 200 nm horizontal bars in the reconstructed images, together with their corresponding mapped square-wave reference profiles. Although only the 200 nm features are presented in Figure 11 for clarity, equivalent profile comparisons (*not shown*) were also performed for the other feature sizes evaluated in this study. Figure 11E and Figure 11F summarise the corresponding IQA metrics obtained for the DIC and PCM datasets, respectively.

The resulting metrics indicate that, for both DIC and PCM imaging, the O-Net models generally exhibit smaller ΔFWHM values than their corresponding U-Net models. Because ΔFWHM represents the difference between the experimentally determined FWHM and the nominal feature dimension [and hence the uncertainty between the measured feature dimensions (based on their intensity profile measurements) and the nominal target dimensions], smaller ΔFWHM values would indicate a closer agreement between the reconstructed feature profile and the specified feature size. An exception is observed for the 170 nm horizontal features, for which the U-Net models produce smaller ΔFWHM values than the corresponding O-Net models in both imaging modalities. However, the Θ-Net models exhibit smaller ΔFWHM values than both U-Net and O-Net for these features. The 140 nm features, however, require particular caution in their interpretation, as these features lie well below the effective resolution limit of the optical system, resulting in the bars and intervening spaces being relatively indistinguishable—an aspect where ΔFWHM becomes particularly indispensable. Consequently, the corresponding IQA metrics should not be interpreted as definitive evidence of reliable resolution of the 140 nm features (despite favourable values obtained in this respect). Nevertheless, the same general comparative trend is observed in this context, with O-Net generally producing lower ΔFWHM values than U-Net, with the exception of the 140 nm vertical features in the SR PCM dataset.

The mean CNR values portray a similar overall trend, with the O-Net models generally producing higher mean CNR values than the corresponding U-Net models, while the Θ-Net models exceed both the U-Net (and O-Net) models in selected cases, such as the 200 nm SR DIC features (<CNR_Θ-Net (200 nm – SR DIC)_> = 1.228; <CNR_U-Net (200 nm – SR DIC)_> = 0.461). Notably, U-Net produces the highest mean CNR values for the 400 nm and 600 nm SR PCM regions of interest, with values of 1.984 and 2.757, respectively.

A similar trend is also observed when the CTF values are considered for the individual feature orientations. Across the assessed feature sizes, the O-Net and Θ-Net models generally yield higher raw CTF values than their corresponding U-Net models, although an exception is observed for the 600 nm SR PCM horizontal features, for which the U-Net model produces a higher raw CTF value (CTF_U-Net [600nm(H) – SR PCM]_ = 0.193). To provide a single orientation-independent measure for each feature size, the CTF values obtained from the horizontal and vertical features were subsequently averaged arithmetically to obtain the corresponding mean CTF. On this basis, the O-Net models exhibit equivalent (or higher) mean CTF values than their corresponding U-Net models across all evaluated feature sizes for both DIC and PCM modalities. Thus, although U-Net produces a higher raw CTF for the specific 600 nm SR PCM horizontal feature orientation, the arithmetic averaging of the horizontal and vertical measurements results in an equivalent overall mean CTF for O-Net at that feature size, with Θ-Net exhibiting a higher mean CTF (<CTF_U-Net (600nm – SR PCM)_> = 0.140; <CTF_O-Net (101ep, 600nm – SR PCM)_> = 0.140; <CTF_Θ-Net (600nm – SR PCM)_> = 0.155).

### 4.2. Hypothesised Fourier-Domain Interpretation of the Trained DNN Models

The inner workings of these assayed DNN models may be attributed partially to their ability to operate in the rear focal plane (Fourier domain) of the microscope’s optical train [34], allowing the resolution of features from learnt convolution (*blurring*), as well as the corresponding transposed convolution (*deconvolution*) kernels. Image formation in the spatial domain (denoted as *j*(*x*,*y*)) may be expressed as the convolution of the *point spread function* (PSF) of the optical train *h*(*x*,*y*) with the impulse response of the linear system *g*(*x*,*y*), which can be characterised by Equation (7) as follows [46]:

*j*(*x*,*y*) = *g*(*x*,*y*)⊛*h*(*x*,*y*) (7)

where ⊛ represents convolution and *h*(*x*,*y*) may be approximated by a Gaussian kernel.

**Figure 12:**
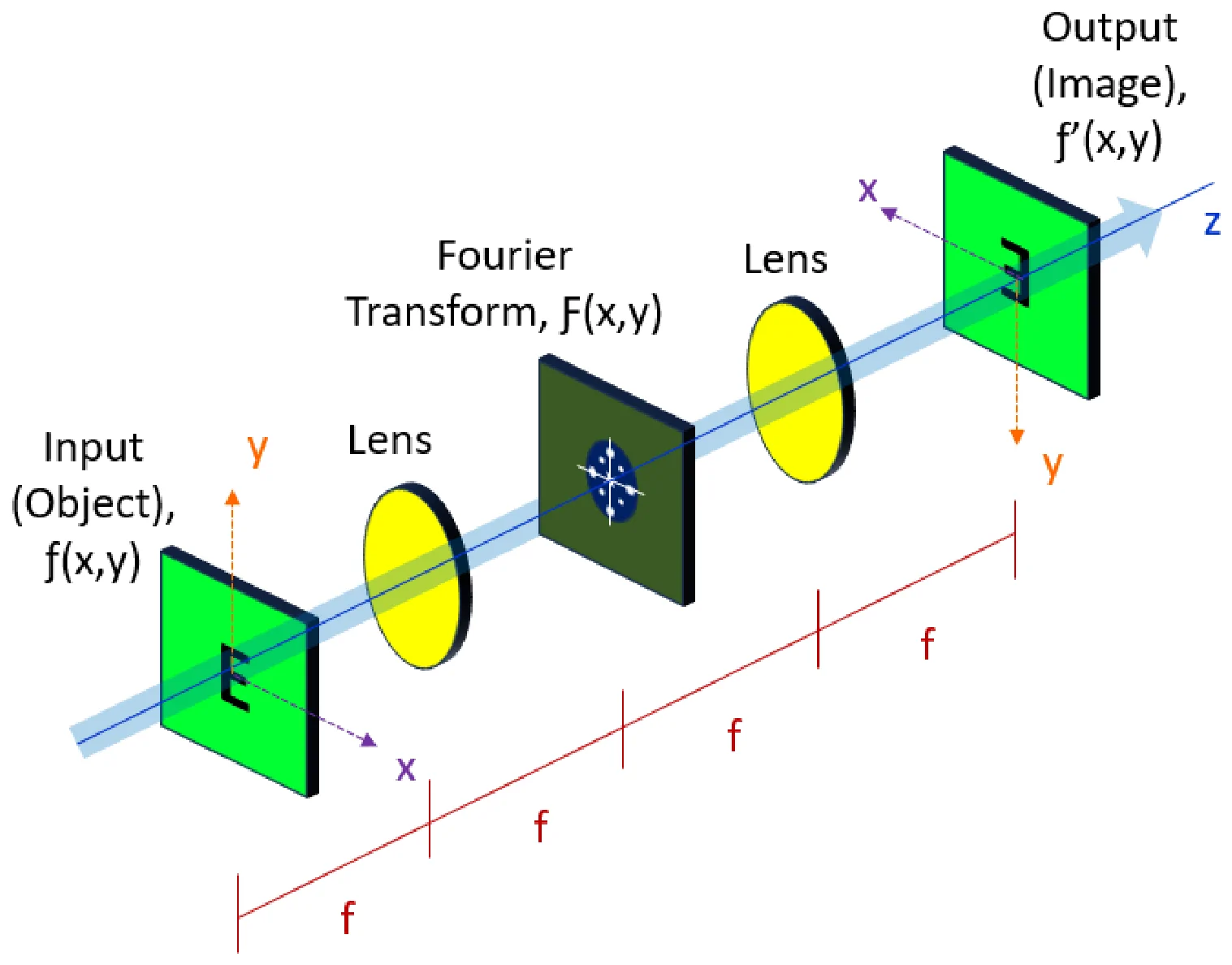
Transmission of light rays through two lenses arranged in a 4*f* optical setup; the image is formed at the image plane (4*f*), while the frequency spectrum is observed at the Fourier plane between the two lenses (2*f*). Higher spatial frequencies (contributed by smaller structures) are generally found at the periphery of the frequency spectrum, while the central maximum corresponds to the lower spatial frequencies (i.e., larger structural features) [2]. The PSF of the optical system would thus also have its FT [the *optical transfer function* (OTF)] [2] projected onto the rear focal plane of the microscope’s objective lens.

It is well-established in the literature [46] that the convolution operation in Equation (4) translates to *multiplication* in the Fourier domain, denoting an aspect that is often explored in *Fourier optics* [47]. When considering the operation of the models in the Fourier (*frequency*) domain, the trained DNN models are thus able to apply a *learnt* conjugate function to extend the Nyquist sampling limit, thereby allowing the resolution of features below the Rayleigh diffraction limit when extrapolated to the real (*spatial*) domain [48]. Interestingly, this does *not* require the models to be trained with frequency spectra acquired in Fourier space, although the perceived interoperability of the models across both the spatial and frequency domains in this respect would enable the resolution of features in both feature spaces. Moreover, this interoperability of the models would also account for their general resilience to *image noise* responsible for varying image SNR levels, since noise is often present as *random* high-frequency components in Fourier space and thus would not be considered a principal component to be integrated into the model weights during the training process. This might also account for why Θ-Net models (being trained over multiple O-Net nodes) generally outperform their O-Net counterparts for lower input image SNRs. To illustrate this, one may consider the optical microscope as a 4*f* optical setup (as depicted in Figure 12), where the real image coupled with its conjugate 2D frequency spectrum/Fourier transform (FT) in the rear focal plane of the microscope’s objective lens may be isolated under differing approaches, i.e., Kohler illumination for the former *vs*. critical illumination with conoscopic examination for the latter [34] (see Supplementary Figure S1 for details).

At this juncture, it would be noteworthy to highlight that the afore-described explanation is intended to provide a possible theoretical framework accounting for the observed differences in reconstruction behaviour, rather than an actual demonstrated representation of the true internal processing mechanisms of models adopting either architecture. As such, the direct analysis of learned filters, intermediate feature representations and network frequency responses should be regarded as essential steps towards validating the proposed explanation.

On a separate note, the 5-layer O-Net model architecture employs a 1 × 1 kernel (stride_x,y_ = 1) in both its convolution and transposed convolution layers, preserving the spatial receptive field dimensions across the layers *without* any consequent down/up-sampling (see Figure 1A for details). A similar approach has been described in the Deep-STORM architecture as well [19], where this is regarded as a 1 × 1 depth-reducing convolutional filter (instead of a raw convolution operation). This is unlike the 7-layer O-Net architecture, which employs a 2 × 2 kernel (stride_x,y_ = 1) (as shown in Figure 1A). In this regard, the model seeks to reduce blur and improve resolution through repeated training cycles aimed at approaching the **Expected** (ground truth) image resolution (with each cycle challenged through a corresponding discriminator network), where the **Source** (input) image (comprising the impulse response convolved with the optical system PSF) may be represented as a set of convolved features (with the PSF being the convolution kernel).

### 4.3. Comparison of Model-Generated Outputs with Other Computational SR Approaches

In the current context, Figure 5B, Figure 6B, Figure 7B and Figure 8B depict the results of resolution enhancement of the **source** images using the widely popular image deconvolution algorithms [Richardson-Lucy (R-L) and Wiener], as well as FISTA and A-Net (a DNN-mediated image SR algorithm developed by [17]). Interestingly, it may be observed from all of these Figures that the deconvolution algorithms used (i.e., R-L, Wiener and FISTA) did *not* provide any significant improvements in the image resolution for both PCM and DIC micrographs when compared with their corresponding **Source** images (acquired under intermediate magnifications of 2.4× and 3.75×). Closer inspection reveals that a potential reason underlying this anomaly might be attributed to the relatively low SNR/CNR of the **Source** images, leading to the inability of these alternative approaches to discriminate between the bars (features) and spaces (background) in the present context. The situation appears to be further exacerbated through the use of A-Net, where the background remains relatively dark, with the features present (if any) being hardly distinguishable from the surrounding extinct background. The SNR/CNR of the input images may thus be deduced to be essential in determining the efficacy of these approaches for image SR, particularly for phase-dependent optical microscopy modalities such as PCM and DIC microscopy. Nonetheless, it should also be emphasised at this juncture that these metrics (CNR, SNR, FWHM, Correlation mean) should **not** be regarded as definitive measures of a model’s ability to recover previously unresolved spatial information, but rather as a means to determine/quantify the relative improvement in the visual/perceptive quality of the model-generated images via feature delineation and an agreement between reconstructed intensity profiles and the known geometry of the test target, thereby facilitating an objective comparison of the respective model outputs and determining which model performs optimally under the various test cases. In addition, the A-Net implementation in the current study employed a pretrained model originally developed for fluorescence microscopy; hence, its performance on the present PMMA test target may not represent the optimal performance attainable following application-specific retraining.

On a separate note, variations in the PSF at different points of the sample (due to slight differences in the mountant refractive index and sample thickness) may also account for these anomalous results as depicted in the R-L/Wiener/FISTA-deconvolved images, since the current parameters applied to these algorithms were based on *iterative testing* to determine the PSF computationally (and not an optical determination of the PSF). Nonetheless, even an empirical determination of the PSF optically would remain subject to potential errors when compared with the *true* sample PSF (due to the aforementioned sample-induced thickness and refractive index variations across multiple RoIs). In this regard, the DNN models proposed in the current study exhibit a significant advantage over conventional image deconvolution operations, as these models can apply learnt variations in the PSF at different points of the sample (attained through repeated training of the models with a large variety of sample images acquired at these different RoIs), thereby accounting for potential deviations in this respect (to a certain extent). Nonetheless, it would also be prudent to mention at this juncture that the alternative methods employed in this study do ***not*** represent exhaustive or universally optimal implementations of the competing algorithms, which were evaluated using the stated parameterisations (as described in Table 2). As such, potential differences in algorithm-specific optimisation, PSF estimation and parameter tuning could influence the performance of the said algorithms on other (untested) image datasets, necessitating any observed superiority of O-Net/Θ-Net over these methods to be interpreted as a comparison under the experimental and computational conditions employed in the present study, rather than as definitive evidence that these architectures universally outperform all implementations of the competing methods under any image SR context.

The findings of the present study have also been demonstrated to be consistent with the broader recent development of computational approaches for label-free SR optical microscopy. Recent studies have demonstrated deep-learning-assisted resolution enhancement in label-free dark-field microscopy [49], including approaches based on U-Net architectures and physics-informed training data, as well as plasmonic dark-field microscopy combined with convolutional neural networks [50]. More broadly, recent perspectives and reviews have highlighted the role of deep learning for computational enhancement and super-resolution [51,52], interferometric methods [53] and other label-free SR approaches [54] as important directions for overcoming or extending the practical limitations imposed by conventional diffraction-limited microscopy. The present work complements these developments by specifically examining computational SR of conventional non-fluorescent phase-dependent microscopy (namely PCM and DIC) using O-Net and Θ-Net architectures in comparison with U-Net, and benchmarking the reconstructed feature dimensions against an independently characterised AFM-calibrated test target. The observed dependence of model performance on the SNR of the corresponding input (**Source**) images further exemplifies an aspect that may be important when comparing computational SR methods across different datasets and optical configurations, since reconstruction performance may depend not only on the choice of the network architecture, but also on the quality and acquisition conditions (*metadata*) of the acquired input image. Recent work on computational SR likewise emphasises the importance of the priors and assumptions incorporated into learned reconstruction methods, reinforcing the need for quantitative physical validation when assessing whether apparent resolution enhancement corresponds to a reliable recovery of spatial information.

### 4.4. Advantages and Limitations of the Assayed Model Architectures

The comparative performance of O-Net and Θ-Net across the two phase-dependent imaging modalities indicates that their relative advantages are strongly dependent on the signal-to-noise characteristics of the input micrograph. For PCM, O-Net demonstrated the more favourable overall performance under the moderately higher SNR acquisition conditions encountered in the present study (for a 2.4× intermediate magnification), producing clearer separation of the target bars and spaces and generally lower ΔFWHM values than the corresponding alternative architectures (U-Net and/or Θ-Net). In particular, the 101-epoch O-Net model provided the clearest reconstruction of the 200 nm and 170 nm PCM features, with the latter representing a substantial extension beyond the calculated 325/329 nm diffraction-limited resolution of the optical system, exhibiting resolution enhancement factors of 1.63/1.65× and 1.92/1.94× respectively. This behaviour is consistent with the relatively high contrast inherently associated with PCM, which provides O-Net with a comparatively informative input from which to reconstruct high-spatial-frequency structural information. However, this advantage is accompanied by an important limitation—when the PCM input SNR is reduced, the capacity of O-Net to distinguish genuine high-frequency structural information from noise becomes progressively compromised. Conversely, Θ-Net, owing to its concatenation of multiple O-Net nodes, appears better-suited to low-SNR PCM inputs. Indeed, under the reduced-SNR conditions produced by the higher intermediate magnification of 3.75×, Θ-Net models generated visibly superior reconstructions to both O-Net and U-Net. Nevertheless, the same sensitivity to small intensity fluctuations that benefits Θ-Net under low-SNR conditions can become disadvantageous when PCM images possess comparatively high SNR, where the Θ-Net architecture may amplify minor pixel-level fluctuations into visible noise and reconstruction artifacts. This limitation was particularly apparent in the high-SNR PCM dataset (Figure 7), in which Θ-Net models generated substantially noisier images and were consequently less suitable than O-Net for SR reconstruction of the target features.

A similar SNR-dependent complementarity was observed for the DIC micrographs, although the balance between the two architectures differed because of the lower intrinsic contrast and pseudo-relief characteristics of DIC images. Under relatively high-SNR conditions, O-Net produced comparatively clean reconstructions with clear delineation of the 200 nm features and generally favourable quantitative image-quality characteristics, demonstrating its ability to exploit the structured intensity information contained within DIC micrographs (Figure 5). The principal limitation of O-Net in this modality was its reduced robustness when the input SNR became sufficiently low that the intensity variations associated with the nanoscale bars approached the noise level. Under these conditions, Θ-Net exhibited a clear advantage: the low-SNR DIC images acquired at the higher (3.75×) intermediate magnification (Figure 6) were reconstructed more effectively by Θ-Net than by either O-Net or U-Net, allowing the individual target features to be more readily distinguished despite their weak input contrast. This suggests that the multi-node Θ-Net architecture may provide greater tolerance to the loss of image information accompanying low-SNR DIC acquisition. Nevertheless, its increased sensitivity to small pixel-intensity fluctuations remains a limitation, particularly for relatively clean DIC images, where unnecessary amplification of minor fluctuations can manifest as reconstruction noise or artefacts (as depicted in Figure 5). Taken together, the results therefore suggest that O-Net is preferable when DIC images are acquired with sufficiently high SNR, whereas Θ-Net provides a potentially more robust alternative when the acquisition conditions impose substantial noise or contrast degradation. Importantly, these observations should ***not*** be interpreted as establishing fixed universal SNR boundaries—the approximate transition values identified in the present datasets (~7.5 for DIC and ~11 for PCM) are *empirically determined* from the present study and may vary substantially depending upon the microscope, camera, illumination conditions, specimen characteristics and training dataset used.

### 4.5. Potential Future Enhancements and Limitations of the Present Study

As previously discussed, the results presented in this study reveal that the smallest nominal target features for which the present quantitative analysis provides evidence of successful reconstruction were 200 nm (for SR DIC) and 170 nm (for SR PCM) for the O-Net-generated images, implying a resolution enhancement factor of 1.65× (or 1.63×) and 1.94× (or 1.92×) for SR DIC and SR PCM respectively, when considering the Rayleigh diffraction limit of 329 nm (or 325 nm). Nonetheless, the 170 nm features in SR DIC (Figure 9F) and 140 nm features in SR PCM (Figure 10I) are only very slightly resolvable, clearly demonstrating the potential of the underlying O-Net models in further overcoming the Rayleigh diffraction barrier to attain image SR. In this light, a future potential improvement to the proposed model architecture might involve determining the shear angle ε introduced by the Nomarski prisms for the SR DIC images and integrating this into the loss function used for training the models. This would serve to potentially alleviate shear bias introduced through translating the objective prism while enhancing model resilience to pseudo-relief fluctuations. This may also reveal further insights into the impact of light polarisation on the model performance, since DIC (being a polarisation-sensitive imaging modality) would likely be affected by features with higher levels of birefringence (such as muscle fibres, collagen fibrils, etc.).

It would also be noteworthy to mention at this juncture that there is presently *no* feasible image SR metric that can be used to objectively quantify the performance of a DNN model in super-resolving non-fluorescent phase-dependent optical micrographs for which there is *no* precedent/ground truth image to compare the generated image against (i.e., there is no empirically validated blinded image SR metric for phase-dependent microscopical analysis) [34]. The Fourier Ring Correlation (FRC) metric ([55,56,57]), which is often cited as an objective and quantitative measure of image resolution, requires two independent images of the same RoI for comparative assessment and can potentially only be used on fluorescent or darkfield images (where the background is totally dark). Due to this, one possible approach to quantify image resolution for non-fluorescent micrographs (as was employed in this study) would involve the use of an intensity profile plot across a defined RoI in the image, and the establishment of a threshold for the relative intensities corresponding to the individual bars/spaces. However, this method will still face a caveat when attempting to resolve structures well below the diffraction limit, such as ≤140 nm in this study (referring to Figure 10H for details). Under such situations, the boundary between the bars and the spaces becomes increasingly blurry as pixel intensity varies in a somewhat gradual manner, making it challenging to accurately infer the specific locations corresponding to either the bars or spaces. This dilemma is further exacerbated through the inclusion of multiple other factors, such as contrast of the input (**Source**) image influenced by varying illumination intensities, camera exposure levels and image gain. 

On a separate note, several additional IQA metrics (such as SQUIRREL [58], decorrelation analysis [59] and single-image FRC metrics [60]) were also assessed on their suitability for analyses of the **Source** (and model-generated) images in the current study. Nonetheless, on further evaluation of the suitability of these metrics for deployment in the current study, numerous constraints were encountered, e.g., decorrelation analysis may be affected by the presence of certain noise types (e.g., Poisson shot noise) that can contribute frequency-dependent fluctuations that are not genuine specimen detail, while single-image FRC requires two noise-independent realisations of the input image to be compared, which cannot be ascertained in the current context since the current PCM and DIC micrographs are RGB images acquired under broadband halogen illumination, while the model-generated outputs may contain correlated details. SQUIRREL, too, cannot be reliably employed in the current study, as it requires the model-generated image to be compared against a super-resolved *ground truth* image to determine if the SR reconstruction is faithful to the acquired ground truth image and if the model has introduced reconstruction discrepancies/artifacts. However, in the present study, the ‘low-resolution’ input (**Source**) images were actually acquired at the cutting edge of optical resolution currently afforded by the microscope’s optics using an objective lens with the highest possible NA of 1.4 (under both PCM and DIC microscopy); hence, there is *no* ‘super-resolved’ ground truth image that can be acquired for use as a direct reference to compare against the model-generated images in this context. In addition, a key consideration for an IQA metric in this respect would also need to address the optically induced artifacts characteristic of PCM and DIC microscopy (i.e., the ‘halo’ and ‘pseudo-relief’ effects respectively). Reformulation of these IQA metrics to circumvent these limitations would thus be essential before they can be successfully deployed for assessment of the said images acquired via either of these phase-dependent microimaging modalities. For this reason, the development of a single *blinded* IQA metric capable of determining the resolution of an acquired/model-generated image remains a prospective avenue for exploration in future studies (should time and resources permit). In a similar regard, more advanced approaches (such as repeated-acquisition analysis, model ensembles and Bayesian uncertainty estimation [29]) could also be explored as potential future extrapolations of computational SR PCM and DIC microscopy to provide additional insight into model reconstruction variability and confidence.

A third possible limitation in the current study relates to the use of the Ashampoo SHARPEN #5 Professional-deconvolved **Source** image for the reference image. Ideally, it would be preferable to acquire a raw DIC/PCM image through purely optical means to be used as the reference image, but this would require the use of an objective with an NA considerably higher than 1.40 (which is not readily accessible in the market currently). For this reason, it is not possible to acquire a separate raw image to be used as the reference image in the present context, necessitating a different (computational) approach (i.e., image deconvolution) to be adopted to circumvent this limitation. Through an assay of the current repertoire of software applications capable of deconvolving an image to recover pixel-level detail, it was found that Ashampoo SHARPEN #5 Professional produced images that closely correlated with their AFM (ground truth) equivalents, while still retaining the characteristic observable aspects of PCM and DIC microscopy (namely the halo and pseudo-relief effects) without introducing additional artifacts, thereby potentially satisfying the requirement for a reference image to be used for further downstream analysis in the current context. This does not, however, suggest that the Ashampoo SHARPEN #5 Professional-deconvolved images are comparable to (or capable of surpassing) an actual independently optically acquired SR PCM/DIC micrograph using a phase objective with a NA much greater than 1.4 (should such an objective be available in the market), with the latter still being regarded as the ‘gold standard’ for image SR. Additionally, it would also be noteworthy to mention at this juncture that these deconvolved images constitute *computationally generated references* rather than experimentally acquired ground-truth images, and thus should ***not*** be regarded as independent measurements of the underlying signal, as they may introduce potential processing-dependent bias into the SNR/PSNR estimates.

On a different note, it will be essential to highlight that the developed models in the current context are also intended for use with the current microscope/camera setup [i.e., a Leica DM4000M microscope (Leica Mikrosysteme Vertrieb GmbH, Wetzlar, Germany) coupled with a RisingCam^®^ (Chengdu Ruishen Technology Co., LTD, Chengdu, Sichuan, China) E3ISPM12000KPA CMOS camera] [34]. Should an alternative microscope/camera configuration be employed, the PSF of the optical system would inevitably change, necessitating a re-training of the models [34]. As such, the use of a single test target (although serving as a calibrated standard for evaluating the SR potential of the models as in the current context) might not be generalisable to *other* samples and optical systems, suggesting the potential extrapolation of the current model architectures to be tested on other optical setups in future studies (should time and resources permit). In the same light, a potential sensitivity analysis employing different wavelengths may also be considered for future downstream studies. Doing this, however, would likely necessitate the use of several interference filters, a monochromator, or several monochromatic laser sources, although this remains outside the scope of the present study.

At this juncture, the significance of appropriate illumination conditions cannot be understated, with potential deviations from Köhler illumination responsible for introducing imaging artifacts capable of adversely affecting model performance in subsequent image processing and SR applications. Furthermore, variations in image contrast, focal quality, background intensity, and specimen preparation exert substantial influence on model prediction/generation outcomes. As such, these parameters should be carefully optimised within image acquisition protocols designed to generate training datasets for the models, prior to their deployment for downstream image processing/SR applications.

Separately, a potential avenue for future exploration will likely refer to the systematic evaluation of model performance across datasets spanning a broad range of signal-to-noise ratios (SNRs). Such SNR-focused studies would incorporate repeated measurements and uncertainty estimates to establish a more rigorous assessment of robustness, extending beyond the low- to moderate-SNR conditions examined here. While the present work has focused on determining a preliminary quantitative SNR threshold based on the measurements attainable within our dataset, this threshold should ***not*** be regarded as conclusive in any sense, but rather as a *foundational* study facilitating more comprehensive investigations in this respect. A more in-depth characterisation in this context would thus involve detector measurements, repeated acquisitions, and controlled noise variations, thereby allowing one to establish the relative contributions of different noise mechanisms and their effects on model performance. In this regard, future efforts in this domain could explore the extent to which SNR influences downstream image processing and SR performance (including the differential impact of the various types of noise on overall model performance), thereby advancing a deeper understanding of the role of noise characteristics in determining model effectiveness for phase-dependent computational nanoscopy applications.

In addition, another potential avenue for future investigation concerns the use of advanced optical modulation strategies to improve the quality and information content of the acquired images prior to computational reconstruction. The present study primarily addresses the computational stage of the imaging pipeline, in which the information contained within PCM or DIC source images is processed by the respective DNN architectures (O-Net and Θ-Net). However, controlled modulation of optical signals during acquisition may provide an additional means of influencing signal detectability and the effective SNR of the acquired images. For example, chaotic modulation of optical transmittance has previously been investigated as a means of enhancing the sensitivity of optical sensing and monitoring biological samples [61], demonstrating that modulation of the detected optical signal can provide additional information for characterising sample-dependent optical responses. In the context of the present work, such approaches could potentially be investigated in conjunction with phase-dependent microscopy to determine whether appropriately designed illumination or detection modulation can improve the acquisition of weak optical signals and thereby enhance the subsequent performance of O-Net, Θ-Net or other computational SR architectures. Systematic investigation across different modulation schemes and source-image SNRs may therefore provide a useful framework for determining whether improvements in optical signal acquisition can complement computational image reconstruction and ultimately improve the robustness and reliability of label-free phase-dependent SR microscopy.

## 5. Conclusions

In the present study, the image SR performance of models trained on two of our developed DNN architectures (O-Net [21] and Θ-Net [22]) was evaluated against U-Net [33] (a model architecture widely utilised in the industry for training models deployed in computer vision and image sensing applications) [34]. To ensure a fair and unbiased assessment, a calibrated test target (adopting the USAF1951 standard of equally spaced bars) was utilised as a benchmark of image resolution, verified under AFM. From the presented results, it is evident that both the O-Net and Θ-Net models performed relatively well, albeit under very different conditions—O-Net excelled where the image SNR was high, while Θ-Net outperformed both O-Net and U-Net models under conditions of low image SNR [34]. These findings suggest that the choice of architecture may be influenced by the quality of the acquired images, with the relative performance of the two models depending on the imaging modality and acquisition conditions. The observed SNR-dependent performance transitions, including the approximate thresholds identified in this study (~7.5 for SR DIC and ~11 for SR PCM), should nevertheless be regarded as empirical and dataset-specific observations pertaining to the current study, rather than (as previously indicated) universally established criteria for selecting one of these DNN architectures.

Quantitative analysis of the reconstructed images, in conjunction with the AFM-characterised test target, indicated that features with nominal widths of 200 nm in DIC and 170 nm in PCM could be distinguished under the conditions examined. These results are promising in the context of computational enhancement of conventional, label-free optical microscopy, but require careful interpretation. In particular, the nominal Rayleigh resolution estimate of 329 nm (calculated using the measured peak wavelength of the broadband halogen illumination source) or 325 nm (derived via a multiwavelength weighted average approach) provides a reference, rather than a complete characterisation of the effective resolution of the imaging system under broadband illumination; the latter is preferentially determined through the employment of multiple interference filters, laser sources, or a monochromator for sample illumination. Furthermore, improved contrast, sharper feature boundaries, or favourable image-quality metrics do not, in isolation, establish that spatial information absent from the original images has been reliably recovered. Accordingly, the present findings support the potential of O-Net and Θ-Net to improve the visibility and quantitative delineation of fine features under the aforementioned experimental conditions, but do not establish a universal resolution limit for either architecture nor do they provide definitive evidence that the 140 nm SR PCM features were reliably resolved (as may be suggested from Figure 10I).

The proposed Fourier-domain interpretation of the architectures’ behaviour should likewise be regarded as a working hypothesis pending direct analysis. Further studies involving larger and more diverse datasets, repeated acquisitions, explicit uncertainty estimates, and independent quantitative measurements will hence be necessary to assess the reproducibility and generalisability of the observed performance and to distinguish genuine recovery of previously unresolved spatial information from image enhancement. Additionally, the present work provides a foundation for future investigations into model performance across a broader and more systematically sampled range of input image signal-to-noise ratios (SNRs), which could help establish more rigorously the extent to which SNR influences model robustness, generalisability, and effectiveness under different imaging conditions. Such studies would also help determine whether the apparent SNR-dependent performance transitions observed here persist across different datasets, specimens, and optical configurations, rather than being specific to the conditions examined in the present study. Finally, as the performance of learned models may depend on the optical system, acquisition conditions, and characteristics of the specimens, model re-training or further validation may be required when these models are transferred to different imaging configurations. With all of these considerations in retrospect, O-Net and Θ-Net exemplify promising DNN architectures for the training of models directed at the computational enhancement of phase-dependent optical microscopy images, facilitating their potential applications in biomedical imaging, materials science, metrology, and quality control, provided that their performance is established against appropriate physical references for each intended application.

## Figures and Tables

**Table 1:** Training hyperparameters as employed in the O-Net and Θ-Net GAN architectures used in the present study.

Hyperparameter	Discriminator	Generator	Overall GAN
Loss function	MAE and binary cross-entropy (ε = 0.001)		
Optimiser	Adam		
Learning Rate	0.0001		
Batch Size	1		
Data Augmentation	No		
Dataset Partitioning (Train:Val ratio)	3944:610		
Random Seed and Run	Yes, training re-initialised after each run of 12 ep/run for 10 runs (120 ep) or 10 ep/run for 16 runs (160 ep) (please see Figure 1 above for total number of ep)		

**Table 2:** Hyperparameters used in the respective alternative image SR protocols. Unmentioned parameters assumed default values as specified in the application (e.g., MATLAB).

Algorithm	Hyperparameters
Richardson–Lucy (R-L) deconvolution	Gaussian PSF (size = 8, σ = 4); 7 iterations
Wiener deconvolution	Gaussian PSF (size = 8, σ = 4); NSR = 0.01
Fast Iterative Shrinkage Thresholding Algorithm (FISTA)	Airy PSF with pupil = 5 nu; λ = 1 nu; axial distance = 0.5 nu; attenuation factor = 3; width/height = 700 px; number of slices = 1; signal intensity = 255; center (cx, cy, cz) = 0.5% 11 iterations; step = 1; λ = 10^−6^; Haar wavelet; scale = 3
A-Net	Mode: tubulin (applies pre-trained model named *ANNA-PALM_tubulin_wf_945_512x512*)

FWST—full width at set threshold. This method is often used to determine the width of adjacent features from their corresponding point spread function (PSF) plots.

## Data Availability

The data presented in this study are available on request from the corresponding author due to privacy concerns.

## Supplementary Materials

The following supporting information can be downloaded at: https://www.mdpi.com/article/10.3390/nano16201334/s1, Figure S1: Orthoscopic and conoscopic examination of a sample of rabbit cerebrum; Figure S2: Emission spectrum of the halogen lamp used (**A**) coupled with values depicting the lateral resolution at different illumination wavelengths (**B**); Figure S3: Architecture for the 5-layer U-Net models as used for image SR in this study; Figure S4: Architecture for the 5-layer O-Net models as used for image SR in this study; Figure S5: Architecture for the 7-layer O-Net models as used for image SR in this study. Reference [62] is cited in the supplementary materials.
