# Supplementary material for: Empirical Validation of SNR-Dependent Deep Learning Architectures for Label-Free Super-Resolution in Optical Microscopy
Source: Nanomaterials (Basel). 2026 Oct 21;16(20):1334. doi: 10.3390/nano16201334 (PMC13708439; doi:10.3390/nano16201334)
Supplement: Supplementary file 1 [file nanomaterials-16-01334-s001.zip › Supplementary Information.pdf]

Supplementary Information

Alterative perspective exemplifying the light microscope as a Fourier-enabled instrument

Further exemplification of the light microscope as a classic 4*f* optical instrument enabling it to receive signals in both the spatial & frequency (Fourier) domains may be depicted in Figure **S1**, where we have attempted to image a specimen using conventional brightfield microscopy & obtain its corresponding Fourier field (acquired under critical illumination):

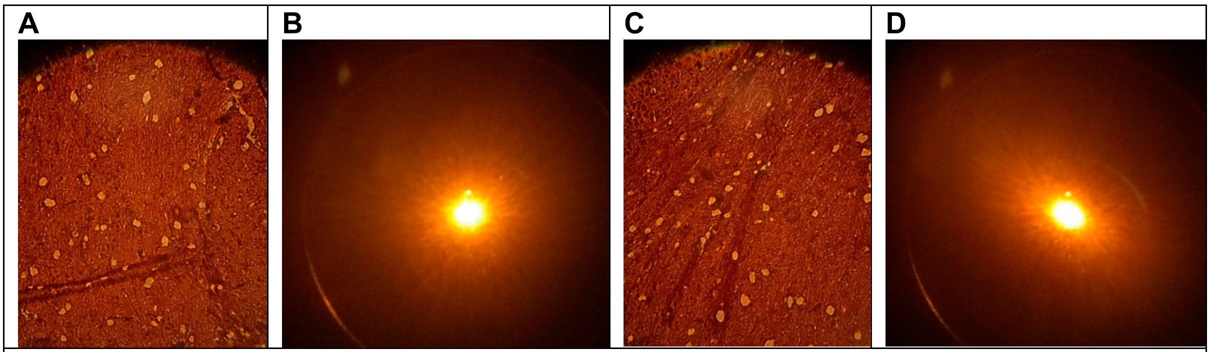

Figure S1: Orthoscopic and conoscopic examination of a sample of rabbit cerebrum. Panels **A** & **C** depict 2 separate RoIs imaged from the same specimen (*rabbit cerebrum section*, *silver stained*). Panels **B** & **D** portray the complex Fourier image field of this same tissue sample with radiating spokes depicting the spatial frequencies observed in the rear focal plane of the microscope's objective lens for *each* of the RoIs shown in panels **A** & **C** respectively. Notably, each point in this pattern (represented by an infinitesimally small point light source) describes a sinusoidal function $I(\boldsymbol{r}) = A\sin(\boldsymbol{k}_{(x,y)} \cdot \boldsymbol{r} + \varphi_0)$ of a specific amplitude *A* and phase φ_0, as well as 2D frequency & orientation vector $\boldsymbol{k}_{(x,y)}$ (direction of propagation of the wave) [62]. Notice the differences in the observed Fourier image fields – features with high spatial frequencies are localized at the periphery of the spectra, while lower frequency features are often clustered in the centroid region. Also as highlighted in [47], the Fourier image fields (**B** & **D**) resemble their respective object fields (**A** & **C**) but are convolved with the Fraunhofer diffraction pattern of the pupil, i.e. a Fourier transform applied to the pupil function. Scale bars are not included in this respect, as this figure is intended to illustrate the underlying OTF behaviour, and is not used for quantitative measurements in the present study.

The emission spectra for the halogen light source (as acquired using a Vernier Instruments SpectroVis Plus ® spectrophotometer) is depicted in Figure **S2** [the peak emission wavelength (λ_{em}) being indicated on the plot].

A

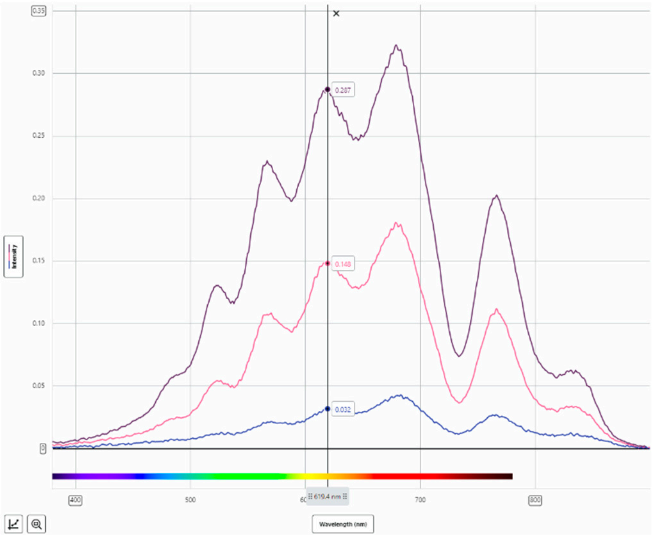

B

λ / nm	N.A.$_{obj}$	N.A.$_{cond}$	Lateral Resolution, d_{xy} (nm)
400	1.4	0.9	212
550	1.4	0.9	292
580	1.4	0.9	308
612.2	1.4	0.9	325
619.4	1.4	0.9	329
654.7	1.4	0.9	347

Figure S2: Emission spectrum of the halogen lamp used (**A**) coupled with values depicting the lateral resolution at different illumination wavelengths (**B**). The peak wavelength in the visible (non-red) spectrum is determined to be **619.4nm**. Computing a weighted average of the visible light wavelengths alone (399.9nm – 700.5nm) based on their relative intensities in the emission spectrum results in a value of **612.2nm**, while taking into account the entire emission spectrum (from 380nm – 899.6nm) results in a weighted average value of **654.7nm**. **B** A table depicting the theoretical lateral resolution (d_{xy}) for different wavelengths (λ) of light used employing the Rayleigh criterion as indicated in Equation (1) previously. When λ = 619.4nm, d_{xy} = **329nm**, but when λ = 612.2nm, d_{xy} = **325nm**. Similarly, when λ = 654.7nm, d_{xy} = **347nm**.

DNN Architectural Details

5-layer U-Net Model Architecture

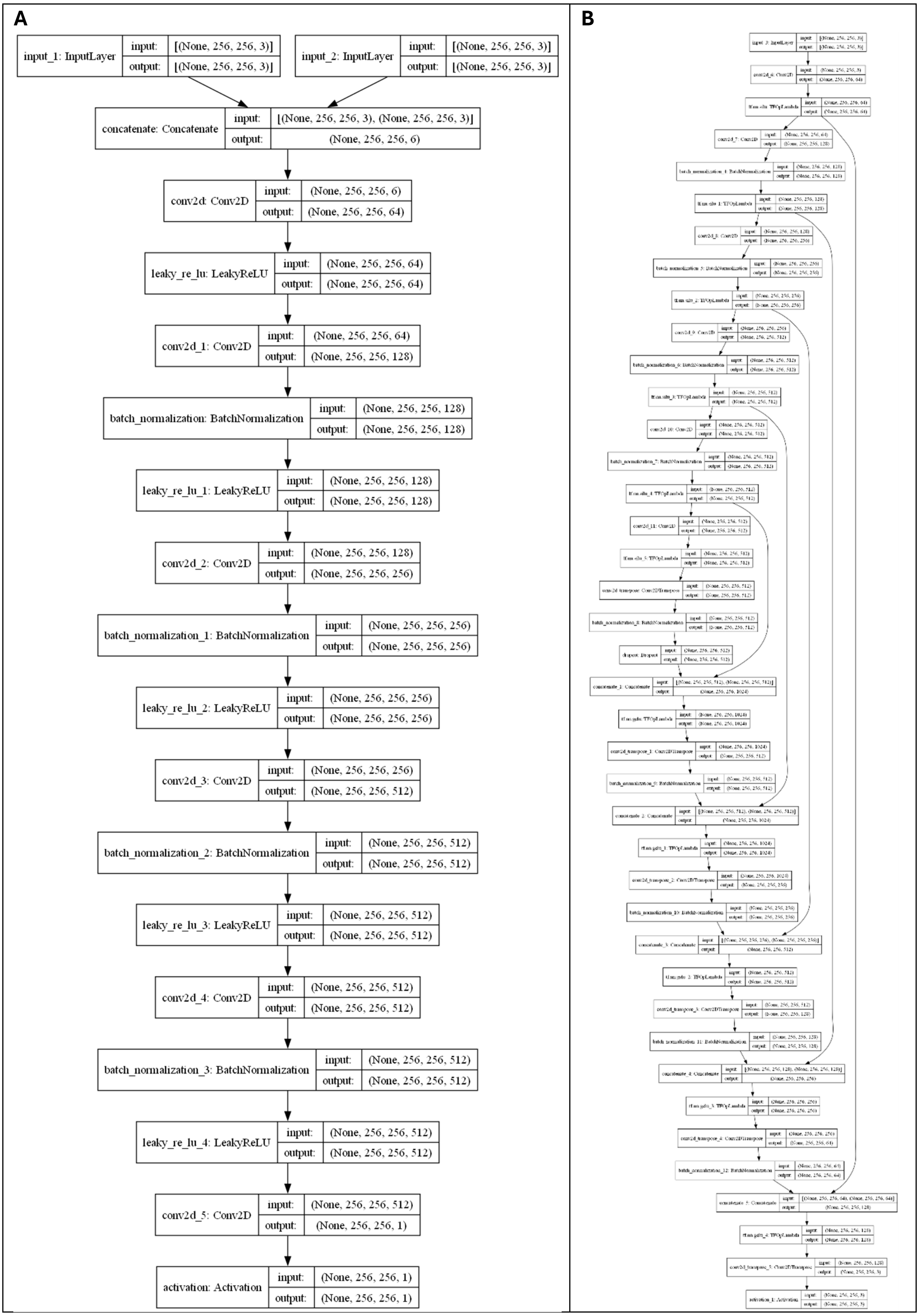

C

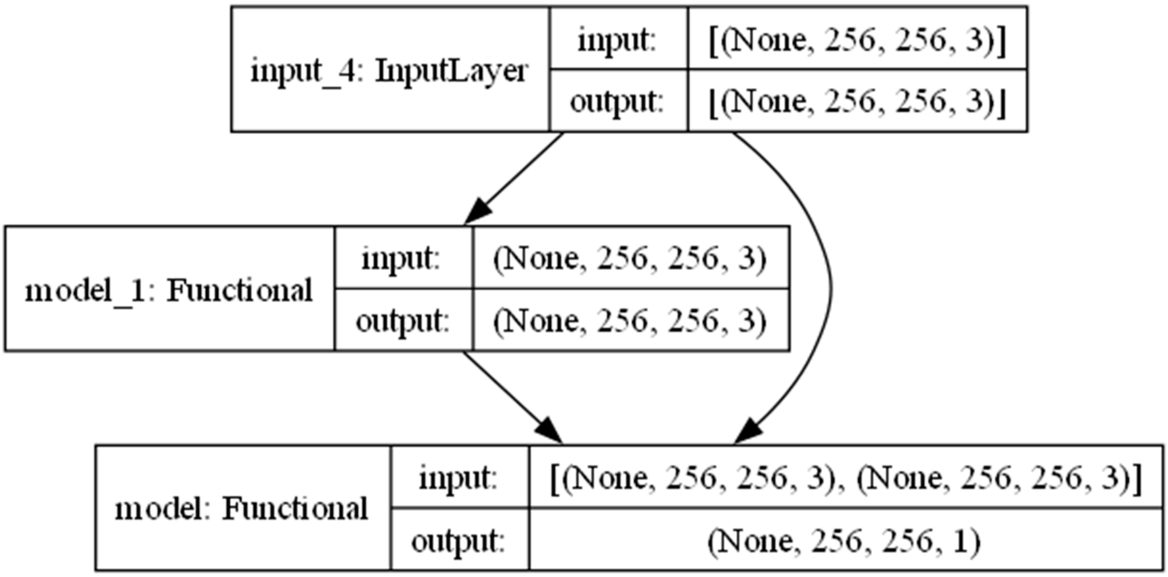

Figure S3: Architecture for the 5-layer U-Net models as used for image SR in this study. **A** Discriminator architecture; **B** Generator architecture; **C** GAN architecture.

5-layer O-Net Model Architecture

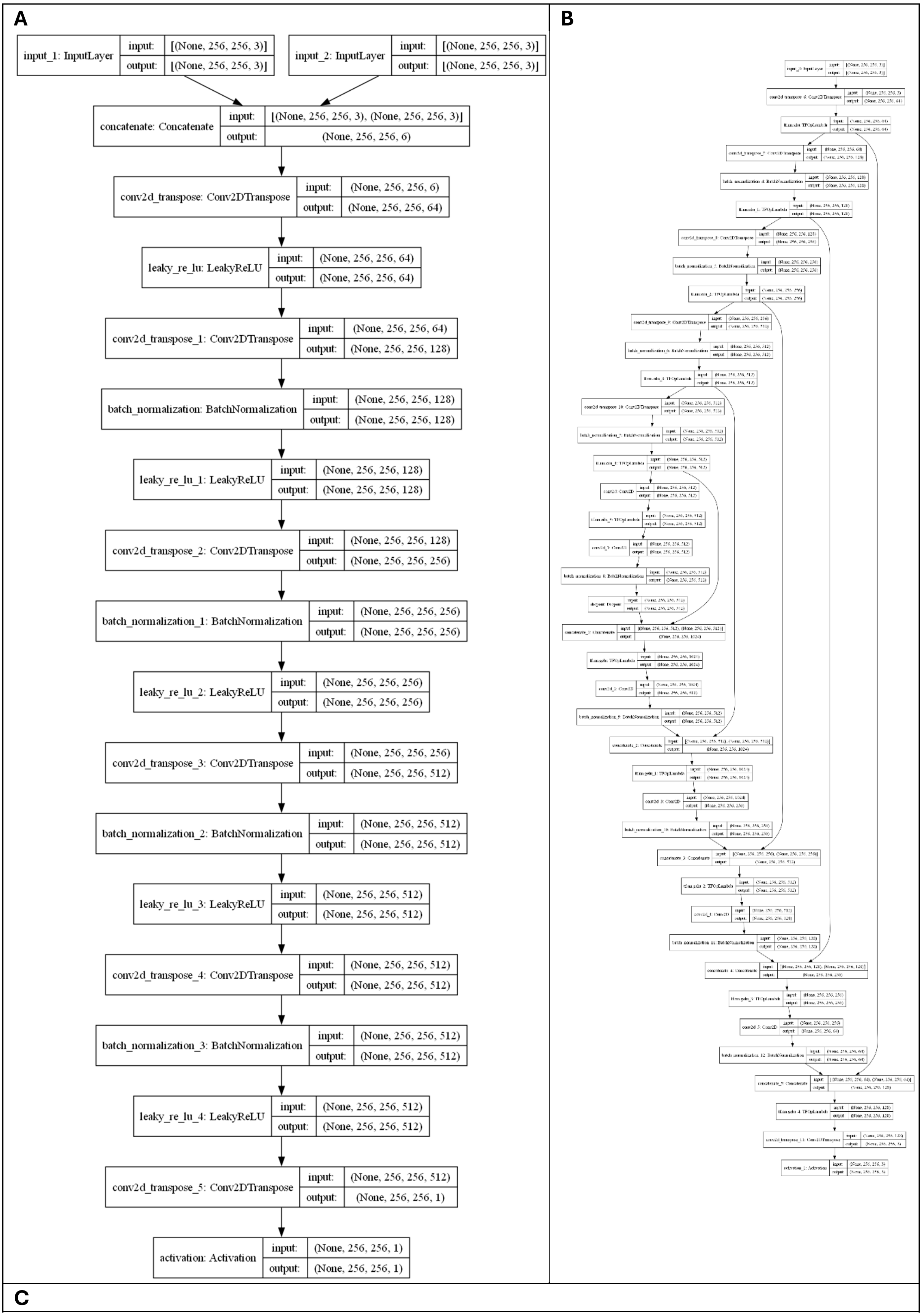

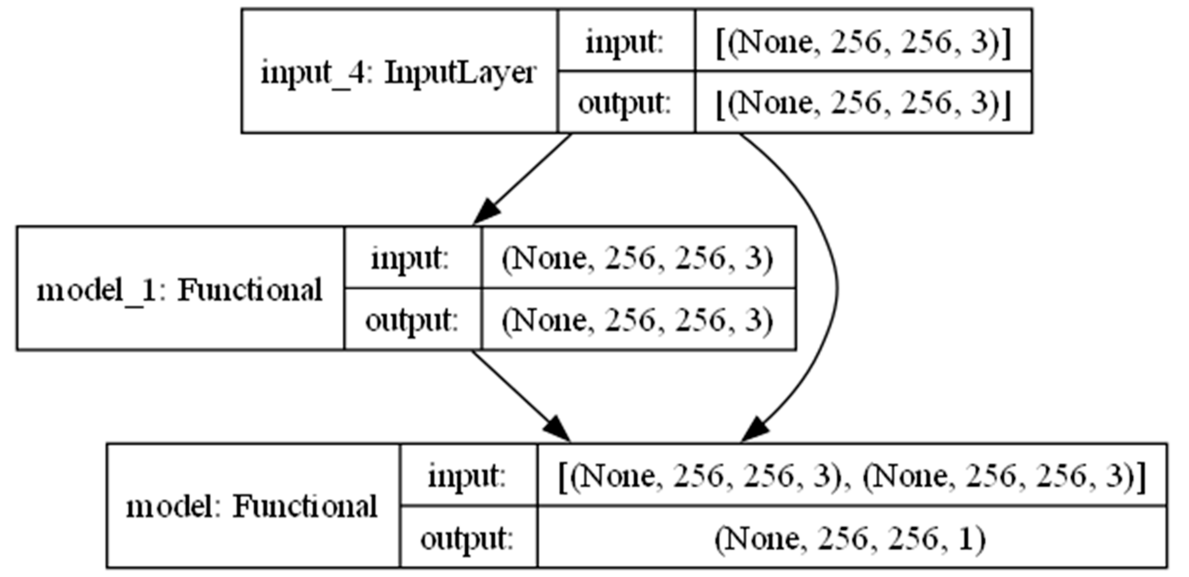

Figure S4: Architecture for the 5-layer O-Net models as used for image SR in this study. **A** Discriminator architecture; **B** Generator architecture; **C** GAN architecture.

7-layer O-Net Model Architecture

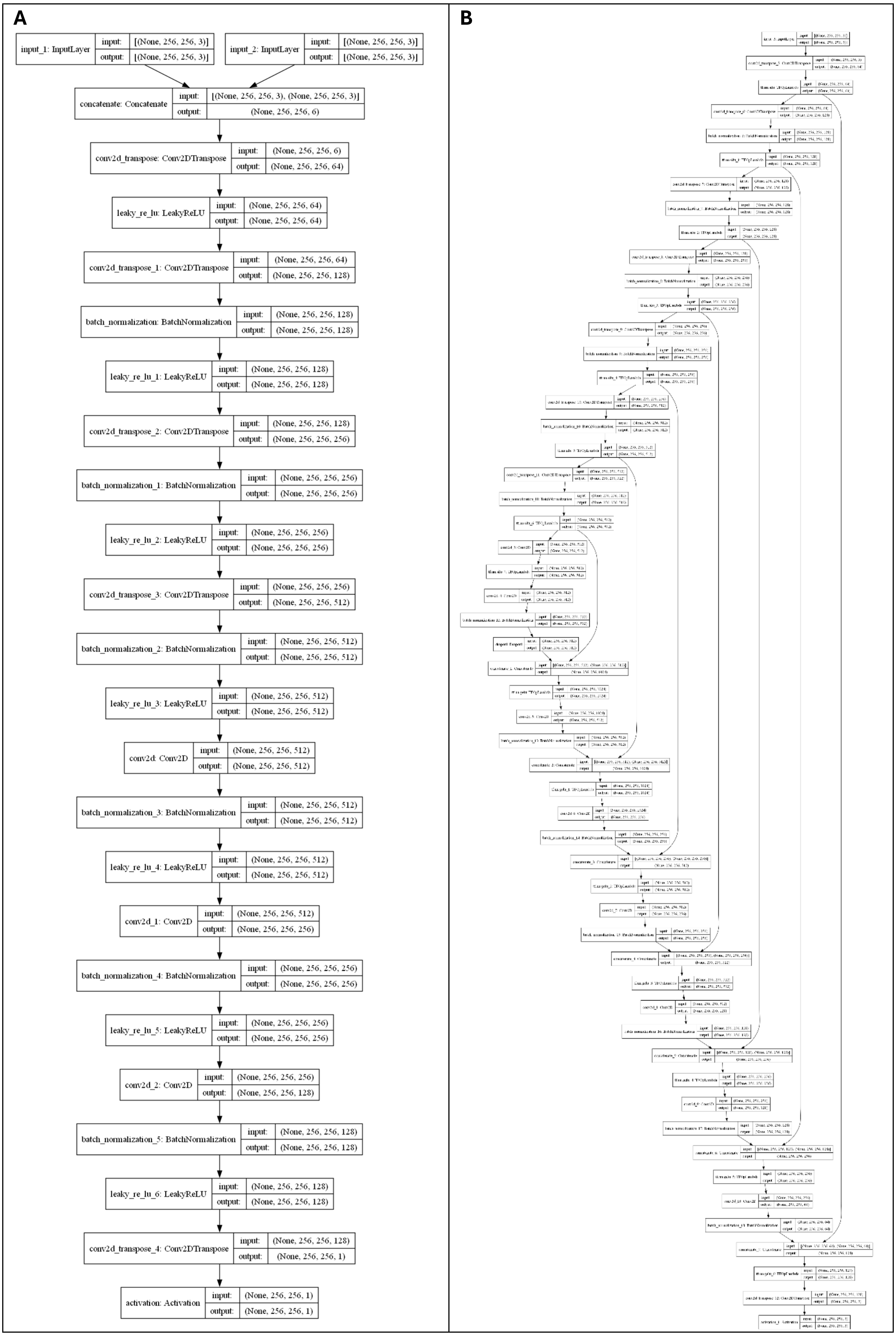

C

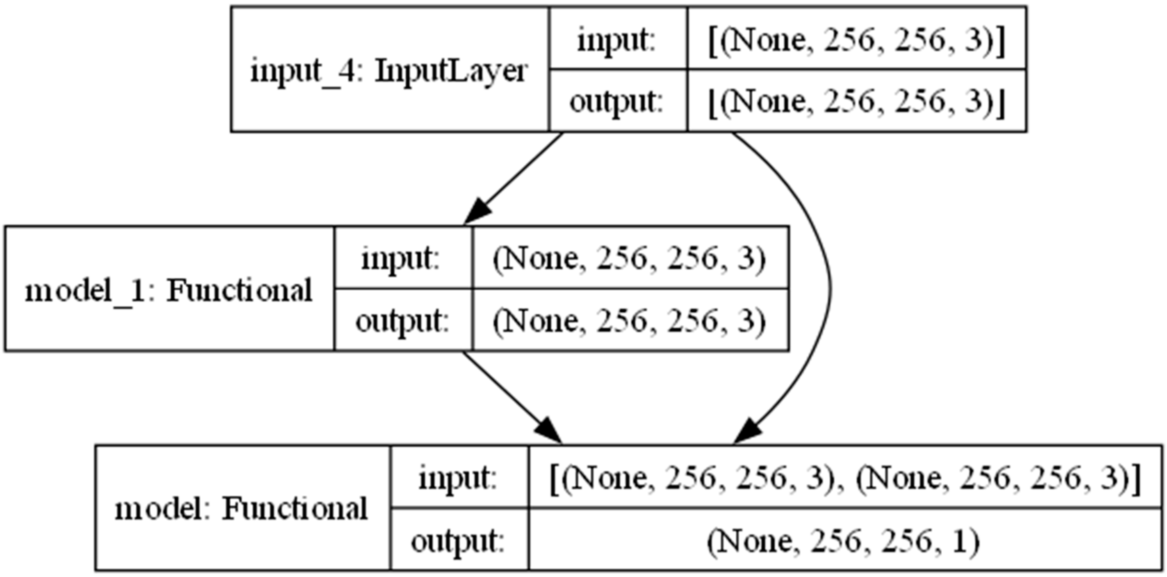

Figure S5: Architecture for the 7-layer O-Net models as used for image SR in this study. **A** Discriminator architecture; **B** Generator architecture; **C** GAN architecture.
